# Disease Severity and Immune Activity Relate to Distinct Interkingdom Gut Microbiome States in Ethnically Distinct Ulcerative Colitis Patients

**DOI:** 10.1128/mBio.01072-16

**Published:** 2016-08-16

**Authors:** Jordan S. Mar, Brandon J. LaMere, Din L. Lin, Sophia Levan, Michelle Nazareth, Uma Mahadevan, Susan V. Lynch

**Affiliations:** Division of Gastroenterology, Department of Medicine, University of California, San Francisco, San Francisco, California, USA

## Abstract

Significant gut microbiota heterogeneity exists among ulcerative colitis (UC) patients, though the clinical implications of this variance are unknown. We hypothesized that ethnically distinct UC patients exhibit discrete gut microbiotas with unique metabolic programming that differentially influence immune activity and clinical status. Using parallel 16S rRNA and internal transcribed spacer 2 sequencing of fecal samples (UC, 30; healthy, 13), we corroborated previous observations of UC-associated bacterial diversity depletion and demonstrated significant *Saccharomycetales* expansion as characteristic of UC gut dysbiosis. Furthermore, we identified four distinct microbial community states (MCSs) within our cohort, confirmed their existence in an independent UC cohort, and demonstrated their coassociation with both patient ethnicity and disease severity. Each MCS was uniquely enriched for specific amino acid, carbohydrate, and lipid metabolism pathways and exhibited significant luminal enrichment of the metabolic products of these pathways. Using a novel *ex vivo* human dendritic cell and T-cell coculture assay, we showed that exposure to fecal water from UC patients caused significant Th2 skewing in CD4^+^ T-cell populations compared to that of healthy participants. In addition, fecal water from patients in whom their MCS was associated with the highest level of disease severity induced the most dramatic Th2 skewing. Combined with future investigations, these observations could lead to the identification of highly resolved UC subsets based on defined microbial gradients or discrete microbial features that may be exploited for the development of novel, more effective therapies.

## IMPORTANCE

Despite years of research, the etiology of UC remains enigmatic. Diagnosis is difficult and the patient population heterogeneous, which represents a significant barrier to the development of more effective, tailored therapy. In this study, we demonstrate the clinical utility of the gut microbiome in stratifying UC patients by identifying the existence of four distinct interkingdom pathogenic microbiotas within the UC patient population that are compositionally and metabolically distinct, covary with clinical markers of disease severity, and drive discrete CD4^+^ T-cell expansions *ex vivo*. These findings offer new insight into the potential value of the gut microbiome as a tool for subdividing UC patients, opening avenues to the development of more personalized treatment plans and targeted therapies.

## INTRODUCTION

Though murine and human studies support the involvement of the gut microbiota in the development and pathogenesis of ulcerative colitis (UC; a common form of inflammatory bowel disease [IBD]), a single causative microbial agent has not been identified and depletion of bacterial diversity remains the primary constant feature of UC gut microbiome dysbiosis (1). Increasingly, disease endotypes have been described among patients within clinically defined chronic inflammatory diseases (2), suggesting that, in the context of immune dysfunction, distinct pathogenic processes may converge upon a common clinical disorder. Since UC pathogenesis is related to gut microbiome composition, we rationalized that factors that dictate the composition and function of these communities may lead to the development of distinct gut microbiome states that function as discrete pathogenic units to deterministically influence immune activation status and disease severity.

Host genetics, diet, and environmental exposures, three factors encompassed by ethnicity, influence both the gut microbiome and UC pathology (3). Indeed, healthy subjects in the United States, Venezuela, and Malawi exhibit a significant relationship between ethnicity and both the composition and function of the fecal microbiota, with diet representing strong selective pressure on the gut microbial assemblage (4). Independently, Frank et al. demonstrated that in a U.S. cohort, IBD risk alleles *ATG16L1* and *NOD2* (associated with autophagy and the host response to microbes, respectively) are significantly associated with gut microbiome β diversity (5). However, a meta-analysis of genome-wide association studies indicated that such UC risk alleles characteristic of Caucasian populations do not confer a heightened risk on ethnically distinct north Indian subjects (6). On the basis of these observations, we hypothesized that distinct pathogenic microbiotas exist within UC patients that covary with both patient ethnicity and disease severity. Given the emerging evidence of gut microbial metabolic dysfunction as a characteristic of immune activation (7), we further postulated that these distinct pathogenic microbiotas exhibit a predictable program of luminal metabolism that induces significantly different degrees of Th2 activation.

## RESULTS

### Interkingdom gut microbiota perturbations are characteristic of UC patients.

**FIG 1:**
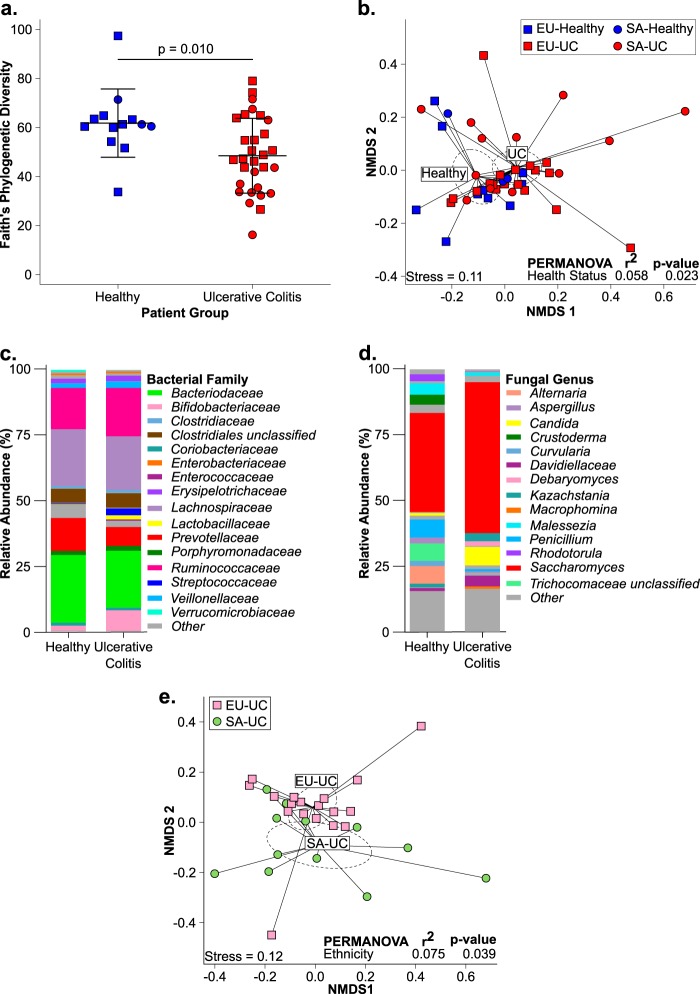
Comparison of healthy (*n =* 13) and UC-associated (*n =* 30) fecal microbiotas. (a) Bacterial α diversity. Horizontal bars represent means ± standard deviations. *P* values were obtained by two-tailed Student *t* test. (b) Bacterial community composition represented by nonmetric multidimensional scaling (NMDS) of pairwise weighted UniFrac distances. Compositional bar plots of the mean relative abundance of bacterial families (c) and fungal genera (d) are shown. (e) Bacterial community composition of UC patients stratified by ethnicity (18 EU UC, 12 SA UC) represented by NMDS of pairwise weighted UniFrac distances. In panels b and e, each dashed ellipse represents the 95% confidence interval for the centroid of each stratification group as calculated by ordiellipse.

Our study population consisted of a cohort of 43 subjects (30 UC patients and 13 healthy subjects) of self-reported European or South Asian (SA) ethnicity (see Text S1 in the supplemental material). Several studies have examined bacterial community composition in fecal samples from UC patients; however, to date, none have examined the mycobiome of adult UC patients. Using parallel, high-resolution bacterial (16S rRNA) and fungal (internal transcribed spacer 2 [ITS2]) biomarker gene profiles, we confirmed that our ethnically restricted UC population exhibited bacterial microbiota dysbiosis consistent with that previously described (1). Compared to healthy subjects, UC patients had significantly reduced α-diversity (*P* = 0.010; Fig. 1a) and were compositionally distinct (permutational multivariate analysis of variance [PERMANOVA]: weighted UniFrac, *R*^2^ = 0.058, *P* = 0.023) (Fig. 1b). Neither fungal α- or β-diversity differed between healthy and UC patients (*P* = 0.523; see Fig. S1a in the supplemental material) (PERMANOVA: Bray-Curtis, *R*^2^ = 0.038, *P* = 0.129; see Fig. S1b), indicating that while profound bacterial depletion is characteristic of the UC gut microbiota, more subtle changes in fungal taxonomy characterize these patients.

A total of 165 bacterial taxa were significantly differentially enriched in healthy participants and UC patients (see Table S1a in the supplemental material). Consistent with previous reports, specific *Bacteroides* and *Prevotella* species and a number of unclassified members of the families *Lachnospiraceae* and *Ruminococcaceae* were among the bacterial taxa most significantly depleted in UC gut microbiotas (Fig. 1c; see Table S1a) (8, 9). UC patients also exhibited enrichment of members of the *Streptococcus*, *Bifidobacterium*, and *Enterococcus* genera (Fig. 1c; see Table S1a), which was validated by independent phylogenetic microarray profiling of these same samples (see Table S1b) and confirms previous reports (8, 9). Only a small number of fungal taxa (*n =* 13) exhibited differential relative abundance (Table S1c). UC patients were depleted of *Alternaria alternata*, *Aspergillus flavus*, *Aspergillus cibarius*, and *Candida sojae* while being significantly enriched in *Candida albicans* and *Debaryomyces* species (Fig. 1d; see Table S1c). Collectively, these data indicate that the UC-associated gut microbiota is characterized by an interkingdom dysbiosis, highlighted by significant expansion of putatively pathogenic bacterial and fungal species, in the context of depleted bacterial diversity.

### UC fecal microbiotas segregate by ethnicity, dominant microbial features, and disease characteristics.

We next addressed our hypothesis that ethnicity is associated with distinct interkingdom fecal microbiota in UC patients. Healthy EU and SA participants exhibited no significant difference in bacterial or fungal α diversity (see Fig. S1C and D in the supplemental material). However, SA-UC patients consistently exhibited less bacterial diversity than either healthy ethnically matched controls or EU UC patients (see Fig. S1c). They also were significantly depleted of fungal diversity compared to the EU UC group (see Fig. S1d), indicating more severe interkingdom microbiome depletion in these patients, though no difference in clinical disease severity between EU and SA-UC patients was observed (see Fig. S1e). Ethnicity was also significantly associated with bacterial, but not fungal, β diversity when all of the participants were considered (see Fig. S1f and g). Because health status was significantly associated with gut microbial composition (Fig. 1b), it represented a potential confounding factor. We therefore repeated PERMANOVA with only UC patients and showed that, while fungal community composition does not exhibit a significant relationship with patient ethnicity (PERMANOVA: Bray-Curtis, *R*^2^ = 0.061, *P* = 0.107), bacterial β diversity does (PERMANOVA: weighted UniFrac, *R*^2^ = 0.075, *P* = 0.039; Fig. 1e), an observation validated by PhyloChip data (see Fig. S1h). Thus, these data indicate that, despite chronic colonic inflammatory disease, ethnicity remains associated with compositionally distinct bacterial communities in the UC gut, though it explains only a small proportion (7.5%) of the observed variation in β diversity across these patients.

**FIG 2:**
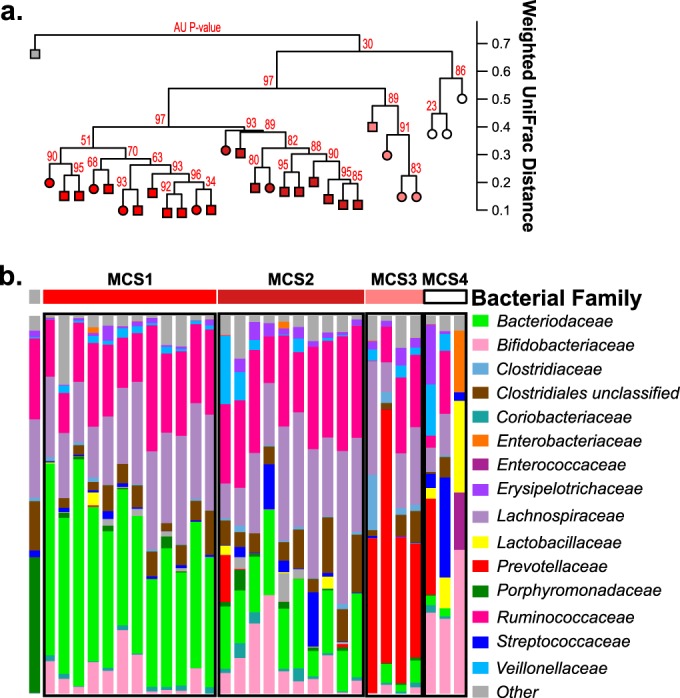
Comparison of fecal bacterial communities among all UC patients (*n =* 30). (a) Hierarchical cluster analysis using pairwise weighted UniFrac distances. Approximately unbiased *P* values (red) computed by multiscale bootstrap resampling. EU UC, squares; SA UC, circles. (b) Compositional plots of bacterial family relative abundance for each UC patient.

Recent pediatric Crohn’s disease studies have demonstrated that patients cluster into subgroups based on patterns of microbial coassociation (10, 11). We next asked whether such patterns exist in our adult UC cohort and relate to patient ethnicity and/or clinical correlates of disease severity. Using hierarchical cluster analysis and multiscale bootstrap resampling, we identified four subgroups of UC patients based on fecal bacterial community composition and termed these microbial community state 1 (MCS1) to MCS4 (Fig. 2). These distinct patient subgroups were confirmed by PERMANOVA with both 16S rRNA sequence and PhyloChip data (see Fig. S2a and b in the supplemental material). MCS distribution differed significantly across ethnicities, with EU UC populations primarily composed of MCS1 and MCS2 while SA UC patients exhibited a relatively equal distribution of all four MCSs (Fisher exact test, *P* = 0.042; see Fig. S2c).

**FIG 3:**
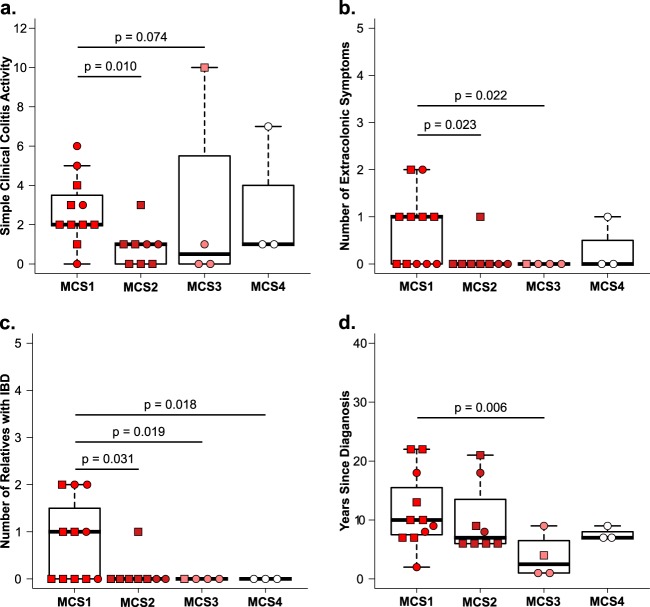
Clinical measurements of UC severity among UC MCSs (11 for MCS1, 8 for MCS2, 4 for MCS3, 3 for MCS4). (a) Simple clinical colitis activity. (b) Number of extracolonic symptoms. (c) Number of family members diagnosed with IBD. (d) Duration of disease. All pairwise comparisons were done with a two-tailed Dunn test. Only *P* values of <0.1 are indicated. EU UC, squares; SA UC, circles.

The clinical relevance of grouping patients on the basis of MCSs was assessed by using an intergroup comparison of clinical disease severity (simple clinical colitis activity [SCCA] index) (12), extracolonic manifestations (arthritis, pyoderma gangrenosum, erythema nodosum, and uveitis), the number of first- and second-degree relatives diagnosed with IBD, and duration (years since UC diagnosis). MCS1 patients exhibited more severe disease with higher median SCCA scores, a significant increase in the number extracolonic manifestations, a greater number of first- and second-degree relatives diagnosed with IBD, and longer disease duration (Fig. 3). Though the number of patients in our study is small, these data provide the first indication that distinct pathogenic UC gut microbiotas exist and are associated with clinical features of disease severity.

### UC MCSs exhibit distinct taxonomic enrichments, metagenomic capacity, and metabolic productivity.

**FIG 4:**
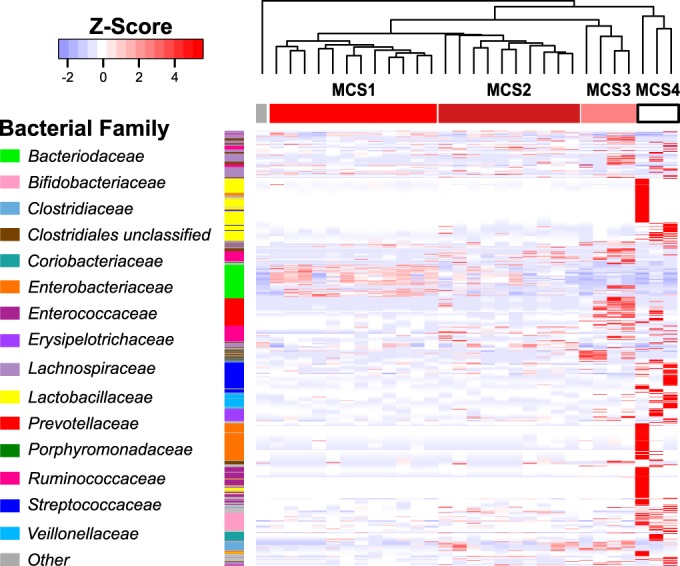
Heat map of bacterial OTUs significantly enriched across UC MCSs. The OTUs shown were identified by Kruskal-Wallis test comparing distributions among UC MCSs (*P* value, <0.05; *q* value, <0.08). Column order is consistent with Fig. 2. Rows are ordered on the basis of phylogenetic relatedness. For visualization, read counts were normalized [log_2_(*x* + 1)] and scaled by row.

The distribution of microbial taxa across the four UC MCSs was assessed to identify specific bacterial and fungal enrichments characteristic of each. Each MCS typically exhibited a distinct dominant bacterial family (MCS1, *Bacteroidaceae*; MCS2, *Lachnospiraceae/Ruminococcaceae*; MCS3, *Prevotellaceae*; MCS4, *Bifidobacteriaceae*) (Fig. 2b and 4). These MCS-specific bacterial enrichments extended beyond the dominant family and were further emphasized when the highest disease severity group (MCS1) was compared to each of the other three groups (MCS2, -3, or -4). Specifically, a majority of the bacterial taxa enriched in MCS1 were members of the *Bacteroides* genus, while the other subgroups were enriched for *Blautia*, *Ruminococcus* (MCS2), *Prevotella* (MCS3), or *Bifidobacterium* (MCS4, generalized linear models, *P* < 0.05) species (see Fig. S2d and Table S2a in the supplemental material). Using the dominant bacterial family as a classifier, we validated the existence of MCS1 and -2 (the two major MCSs in EU UC patients) in two publicly available UC microbiota data sets obtained from patients primarily of European descent (see Fig. S3) (9, 11), indicating that these MCSs are not exclusive to our study but exist in UC patient populations nationwide. Mycologically, *C. albicans* and *Debaryomyces* species were most highly enriched in MCS1 patients compared to each of the other three MCSs (generalized linear models, *P* < 0.05) (see Table S2b), indicating that interkingdom gut microbiome expansion of *Bacteroides* species, *C. albicans*, and *Debaryomyces* species is associated with more severe UC disease.

To identify microbiota-derived pathways and products characteristic of each MCS that may modulate the host immune response and contribute to clinical disease severity, we performed *in silico* metagenomic predictions in parallel with broad-spectrum gas and liquid chromatography mass spectrometry of fecal samples. Phylogenetic investigation of communities by reconstruction of unobserved states (PICRUSt; http://picrust.github.io/picrust/) (13) was used to predict bacterial functional capacity. Presently, this algorithm cannot be used to predict fungal community function. Predicted metabolic capacity varied significantly by MCS (PERMANOVA: Bray-Curtis, *R*^2^ = 0.384, *P* = 0.002). A total of 144 bacterial KEGG pathways discriminated MCS1 to -4, including those involved in amino acid and lipid biosynthesis and metabolism (Kruskal-Wallis test, *q* < 0.0006) (see Table S3a and Fig. S4 in the supplemental material). Specifically, differential enrichment of glycerolipid, fatty acid, inositol, and multiple amino acid metabolism pathways, including phenylalanine, tyrosine, tryptophan, glutamate, and glutamine, differentiated these groups (see Table S3a and Fig. S4). We also generated functional predictions for MCS1 and -2 stool samples from the studies of Morgan et al. and Gevers et al. (9, 11). A total of 121 KEGG pathways were differentially enriched between MCS1 and MCS2 in our study; of these, 74 (61.2%) also discriminated MCS1 from MCS2 in both the data sets of Gevers et al. and Morgan et al., indicating a high degree of conserved microbial function associated with MCS1 and -2 across multiple independent studies (see Table S3b).

**FIG 5:**
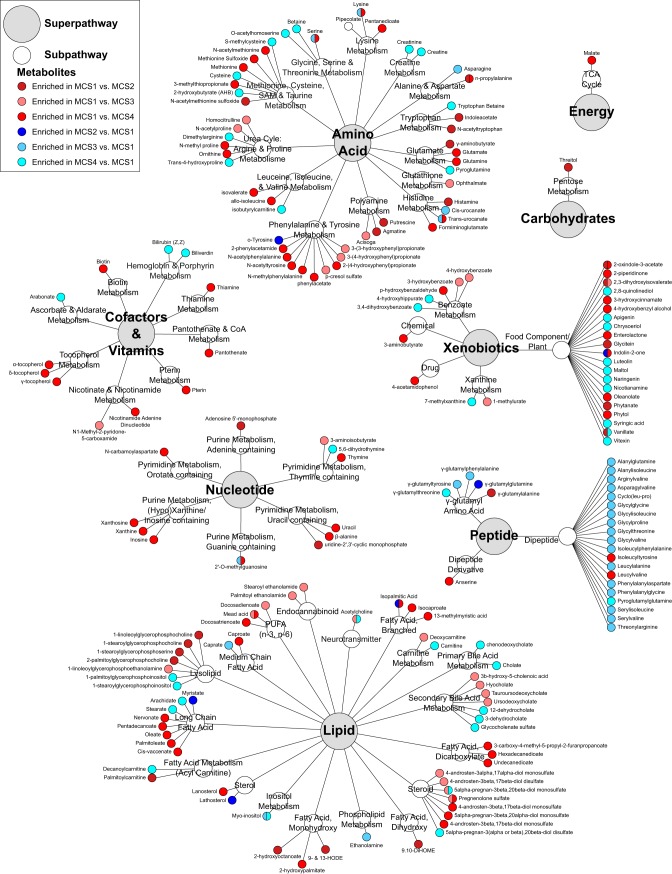
Fecal metabolites significantly differentially enriched among UC patients classified as MCS1, MCS2, MCS3, or MCS4 on the basis of pairwise Welch *t* tests (*P* value, <0.05).

**FIG 6:**
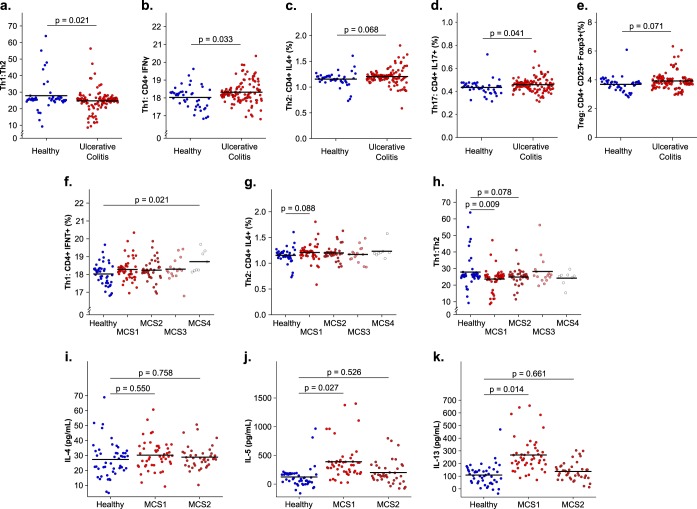
*In vitro* human T-cell activity following coculture with autologous DCs coincubated with sterile fecal water. Panels: a, Th1-to-Th2 ratio; b, Th1 frequency; c, Th2 frequency; d, Th17 frequency; e, regulatory T-cell frequency (48 healthy, 116 UC). Comparisons of the Th1 frequencies (f), Th2 frequencies (g), and Th1-to-Th2 ratios (h) of healthy and UC MCSs are shown (48 for healthy, 48 for MCS1, 40 for MCS2, 16 for MCS3, and 8 for MCS4). Concentrations of IL-4 (i), IL-5 (j), and IL-13 (k) in cell supernatant following coculture of human T cells with autologous DCs challenged with sterilized fecal water from healthy participants and MCS1 and MCS2 patients are shown (48 for healthy participants, 48 for MCS1 patients, and 40 for MCS2 patients). Data were generated from four (a to h) or two (i to k) replicate experiments with DCs/T cells obtained from two anonymous PBMC donors. Horizontal bars (mean fitted values for each group) and *P* values were determined by linear mixed-effect modeling (see Materials and Methods). *P* values of <0.1 are indicated.

We hypothesized that the predicted functional differences across MCSs would be manifested as distinct programs of luminal metabolism, particularly since the majority of the pathways that differentiated these communities were involved in amino acid and lipid metabolism. Indeed, each MCS exhibited significantly distinct metabolic programs (PERMANOVA: Canberra, *R*^2^ = 0.209, *P* = 0.004) that were significantly related to both the fecal microbiota present (Mantel test, *r* = 0.38, *P* < 0.0001) (see Table S4a) and its predicted metagenome (Mantel test, *r* = 0.21, *P* < 0.008) (see Table S4a). We were particularly interested in those luminal metabolites that discriminated the more severe MCS1 from each of the remaining MCSs. Of the 805 metabolites detected across all of the samples, 207 exhibited significant inter-MCS differences in relative concentration (Welch’s *t* test, *P* < 0.05) (Fig. 5; see Tables S4b to d). Compared to MCS groups with lower disease severity, MCS1, as our *in silico* predictions suggested, was significantly enriched for ophthalmate (a biomarker of increased oxidative stress and depleted glutathione) (14), oxidative-stress-inducing putrescine (15), proinflammatory *p*-cresol sulfate (16), 9-hydroxyoctadecadienoic acid and (9-HODE) and 13-HODE (a proinflammatory, leukocyte-recruiting monohydroxy fatty acid) (17, 18), and 9,10-dihydroxyoctadecanoic acid (9,10-DiHOME; a neutrophil-recruiting, cytotoxic dihydroxy fatty acid) (19), as well as bioactive lysolipids involved in leukocyte activation (Fig. 6; see Table S4b to d) (18, 20). In contrast, lower disease severity MCSs (MCS2, -3, and -4) were enriched for a range of potentially protective dipeptides (including anti-inflammatory alanyl-glutamine) (21, 22), γ-glutamyl dipeptides indicative of improved oxidative stress coping mechanisms (23), and antioxidant immunosuppressive *myo*-inositol (24, 25). These observed differences in gut luminal metabolic programming between MCSs associated with high and low UC severities indicate the existence of putative mechanisms to control inflammation in patients with less severe disease.

### T-cell activity *in vitro* is related to MCS and health status.

Recent studies have demonstrated that specific gut microbiome-derived metabolites influence Th2 responses (7) and, independently, that proinflammatory cytokine production by T-helper cell populations, including Th2 cells, is a characteristic of UC (26). We therefore hypothesized that the luminal milieus associated with distinct MCSs differentially influence CD4^+^ T-cell activation in a manner consistent with disease severity. To assess this, we developed an *ex vivo* assay involving coincubation of human dendritic cells (DCs; obtained from healthy donors) with filter-sterilized fecal water prepared from study participants’ feces. DCs were then cocultured with autologous CD4^+^ T cells prior to analyses of T-cell phenotypes and cytokine productivity. Compared to healthy participants, UC patients exhibited a significant reduction in the ratio of Th1 to Th2 cells, significantly increased numbers of both Th1 and Th17 cells, and trends toward increases in both T-regulatory and Th2 cell populations (linear mixed effects, *P* < 0.05) (Fig. 6a to e). CD8^+^ T-cell subsets did not differ significantly between healthy participants and UC patients (data not shown). These findings suggest that luminal microbial products captured in sterile fecal water contribute to UC by inducing a Th2-skewed expansion of CD4^+^ T-cell populations.

Having demonstrated the Th2-skewing effect of UC-associated fecal water, we next asked if this immune response varied on the basis of MCS and associated differences in symptom severity, focusing specifically on Th1 and Th2 populations. With the exception of a minor significant increase in Th1 populations in response to MCS4 fecal water, no significant differences in overall Th1 or Th2 cell populations were observed between MCS groups and controls (Fig. 6f and g). However, when the Th1-to-Th2 ratio was calculated for each group, the MCS1 group exclusively exhibited a significantly lower Th1-to-Th2 ratio compared with healthy controls (Fig. 6h). Of note, no difference in the Th1-to-Th2 ratio was observed when UC patients were compared on the basis of ethnicity (EU UC versus SA UC, see Fig. S5 in the supplemental material), providing evidence that patient ethnicity alone is not responsible for the altered T-cell activity observed *ex vivo*. Furthermore, when considering the two MCSs demonstrating the greatest difference in disease severity (MCS1 and MCS2), only MCS1 fecal water significantly increased secretion of Th2-associated cytokines compared with healthy controls (Fig. 6i to k). These *ex vivo* data provide evidence that compositionally and metabolically distinct UC microbiotas are capable of differentially influencing CD4^+^ T-cell populations in a manner consistent with UC disease severity.

## DISCUSSION

Heterogeneity among UC patients is poorly understood and represents a significant barrier to more effective therapy. Colitis development necessitates microbial involvement, and gut microbiome dysbiosis is characteristic of adult UC patients, but while genetic, therapeutic, and environmental factors are related to UC bacterial β diversity, they explain a small proportion of the observed variation in these microbial communities (5, 9). Microbial species engage in inter- and intraspecies interactions that dictate coassociated microbes and their physiology (27, 28). For example, *C. albicans* coaggregates with specific bacterial species in the oral microbiota, facilitating more robust, stress-resistant mixed-species biofilms (27). In turn, the products of these coassociated bacteria induce a physiological shift toward unicellular morphology in *C. albicans* (27). Similarly, because of metabolic cross-feeding, *Streptococcus gordonii* facilitates coassociation with *Fusobacterium nucleatum* (28). Hence, we rationalized that, under the proinflammatory conditions of the colitic gut, distinct patterns of pathogen coassociation occur whose composition and function are relatively conserved across patients and related to immune activation and disease severity. Our data support the existence of four distinct UC MCSs that differ significantly in their prevalence along ethnic divides. Internal and external validation confirmed the existence of the predominant microbiota states, indicating that, despite inherent patient variability, treatment regimens, and geography, conserved patterns of pathogenic microbiota coassociation exist across UC populations within the United States. To improve our understanding of the progression and development of these MCSs, it will be important for future studies to investigate UC patient factors, be they temporal, clinical, genetic, or environmental, that directly drive the microbiome toward these differential microbial states.

Of the four MCSs identified in our study, MCS1 represented the most ill patient group, implicating the composition and metabolism of MCS1 in enhanced immune activation and increased disease severity. MCS1 characteristically exhibited expansion of *Bacteroides* species, which can produce enterotoxin previously associated with UC, stimulate interleukin-8 (IL-8) and tumor necrosis factor alpha (TNF-α) secretion in intestinal epithelial cells, and intensify colitis symptoms in a murine model of UC (29–31). MCS1 patients also exhibited the greatest expansion of *C. albicans* and *Debaryomyces* species. Gut microbial expansion of these fungal species has also been described in adult and pediatric Crohn’s disease, as well as pediatric IBD (Crohn’s disease and UC patients combined) (10, 32, 33). Together with our study, these data indicate that expansion of *Saccharomycetales* fungi in the context of depleted bacterial diversity is a consistent feature of IBD in pediatric and adult populations. Whether *C. albicans* directly influences UC pathology in patients in our study is unclear. However, gastrointestinal colonization by *C. albicans* impairs gastrointestinal healing in both UC patients and a murine model of UC and can induce a Th2 response following gastrointestinal infection of mice with antimicrobial-depleted gut microbiota diversity (34, 35).

The MCS2 subgroup was enriched for both *Blautia* and *Ruminococcus* species, which together may produce anti-inflammatory short-chain fatty acids (36–38). *Prevotella* species (enriched in MCS3) are capable of suppressing lymphocyte activity, while *Bifidobacterium* species (enriched in MCS4) can reduce the production of both IL-8 and TNF-α in intestinal epithelial cells (39, 40). It should be noted that one patient in our study, who demonstrated a dramatic enrichment of *Porphyromonadaceae* (Fig. 2; see Fig. S2a and b in the supplemental material), was not classified as having one of the four main MCSs identified here and, though removed from our analysis, may represent an additional, clinically relevant MCS that, given additional patient enrollments, future studies may further characterize and draw conclusions from. Though confirmation that the MCSs identified in our study are also present in independent UC microbiome studies indicates the relative durability of these microbial states, their long-term stability cannot be assessed in cross-sectional studies. It is likely that these MCSs represent discrete points along a nonlinear continuum of pathogenic microbial successional states that relate to disease progression and severity, similar to the microbial gradient identified by Gevers et al. in pediatric Crohn’s disease (11). Though these cross-sectional studies are informative, more expansive, longitudinal studies are necessary to determine the natural history of the gut microbiome in UC development and progression.

While interkingdom microbial taxonomic states represent an economical means to stratify patients in large studies, the functional capacity and productivity of these compositionally discrete pathogenic microbiota are paramount to dictating host immune responses and clinical disease severity. Indeed, in our study, programs of metabolic productivity idiosyncratic to the predicted pathways encoded by bacteria present in each MCS were identified. In particular, 9-HODE, 13-HODE, 9,10-DiHOME, and lysophosphatidylcholines (significantly enriched in MCS1) can increase leukocyte recruitment and proinflammatory cytokine secretion (17–20). Soluble epoxide hydrolase inhibitors, which prevent 9,10-DiHOME formation, attenuate UC in both chemical and genetic murine models (41), underscoring a potential role for these oxylipins as contributors to more severe disease and that treatments inhibiting their production may be especially efficacious in this specific patient subgroup. In addition to enrichment of leukocyte chemotactic metabolites, MCS1 patients also had high fecal concentrations of *p*-cresol sulfate, a microbe-derived metabolite (42), and putrescine, both of which can stimulate a leukocyte oxidative burst (15, 16). Consistent with these observations, ophthalmate was also enriched in MCS1 patients, indicative of greater oxidative stress due to low or depleted levels of reactive oxygen species (ROS) quenching glutathione (14). While the metabolome of high disease severity MCS1 indicated conditions of high oxidative stress, that of UC MCSs associated with lower disease severity (MCS2 to -4) exhibited an increased capacity for ROS quenching due to enhanced γ-glutamyltransferase activity indicated by enrichment of γ-glutamyl amino acids (critical for maintaining glutathione levels) and high concentrations of superoxide scavenging *myo*-inositol (23, 24). Metabolic signatures indicative of immunosuppressive activity, such as enrichment of anti-inflammatory dipeptides (i.e., alanyl-glutamine) and *myo*-inositol (both of which decrease the expression of proinflammatory cytokines and reduce leukocyte recruitment in animal models of colitis) (21, 22, 25), were also observed in MCS2 to -4 with lower disease severity. This suggests that the specific metabolic productivity associated with each MCS may govern host immune activity and resulting differences in UC severity.

MCS-associated luminal products, which include host- and/or microbe-derived immunomodulatory metabolites, provide a multifaceted mechanism by which a pathogenic gut microbiota may influence host physiology and dictate clinical disease severity. Though pathogen-associated molecular patterns (PAMPs) have traditionally been considered paramount to driving host immune responses to microbes, emerging data in the field of immuno-metabolism indicate that microbe-derived metabolites are equally effective in dictating immune cell phenotypes. In addition to the established direct immunomodulatory activity of microbe-derived metabolites such as short-chain fatty acids or *p*-cresol sulfate (16, 38), recent studies have demonstrated that the gut microbiota-associated metabolites taurine, histamine, and spermine comodulate NLRP6 inflammasome signaling, epithelial IL-18 secretion, and downstream antimicrobial peptide production (43). Indeed, our data suggest that specific programs of microbe-derived metabolism in combination with an array of PAMPs presented by pathogenic bacteria and fungi in the distal gut of UC patients serve as effective drivers of immune dysfunction related to UC disease severity. Support for this concept comes from our demonstration *ex vivo* that sterile fecal water from the most severely ill MCS1 patients induced the greatest degree of Th2 skewing in T-cell populations and associated cytokine production, a feature not observed among the other subgroups with less severe disease. While this observation does not directly implicate the microbiome as a causative agent of UC, it does provide evidence of the ability of the microbiome to perpetuate the inflammation and symptoms associated with UC in a manner specific to microbiota composition. This finding also indicates that the Th2 skew traditionally considered characteristic of UC patients (26) is not a consistent finding across our cohort and may, in fact, be driven by the most severely ill patients in UC cohorts (i.e., MCS1). Whether or not different inflammatory phenotypes present among UC patients select for phenotype-maintaining microbes or are the result of initial, discrete dysbioses remains to be addressed. Regardless, this raises the possibility that distinct immunological features not examined in this study characterize patients with lower disease activity and distinct gut MCSs. Future larger studies will be important in further characterizing the potential immunomodulatory contributions of theses MCSs while confirming the observations presented here. Hence, therapies tailored to the specific microbial, metabolic, and immune dysfunctions exhibited by UC patient subgroups may prove a highly efficacious strategy for more effective treatment of this disease.

## MATERIALS AND METHODS

### Fecal sample collection and nucleic acid isolation.

Stool samples were collected from healthy participants and physician-diagnosed UC patients of either EU or SA ethnicity by using a standardized protocol. See Text S1 in the supplemental material for details of patient enrollment criteria. Fecal DNA was extracted with a combination of bead beating and the commercially available QIAamp DNA Stool kit (catalog no. 51504; Qiagen, CA). For details of the procedure, see Text S1 in the supplemental material.

### Bacterial 16S rRNA profiling.

Total DNA extracted from fecal samples was used as the template for 16S rRNA gene amplification (in triplicate) with barcoded primers targeting the V4 region as previously described (44). Sequencing libraries were created as previously described (44). See Text S1 in the supplemental material for more detail. Full-length 16S amplicons were also generated and hybridized to the G3 16S rRNA PhyloChip (Affymetrix, CA) as previously described (45). For further details, see Text S1 in the supplemental material.

### Fungal ITS2 library preparation.

ITS2 sequencing libraries were created with triplicate PCR amplicons per sample. For details, see Text S1 in the supplemental material.

### 16S and ITS2 library sequencing

Purified sequencing libraries were analyzed with a Bioanalyzer (Agilent), quantified with the Qubit HS dsDNA Assay kit (Invitrogen), and sequenced with an Illumina MiSeq platform and MiSeq Control Software v2.2.0 according to the manufacturer’s instructions (Illumina). FLASH v1.2.7, QIIME 1.8, and usearch software packages were used for sequence read quality filtering, operational taxonomic unit (OTU) picking, and OTU table generation (46–48). For specific details, see Text S1 in the supplemental material.

### Predicted community metagenome analyses.

PICRUSt (http://picrust.github.io/picrust/) was used to generate *in silico* bacterial metagenomes by using 16S rRNA data (13). For details, see Text S1 in the supplemental material.

### Metabolome profiling.

To profile fecal metabolites, >200 mg of each frozen stool sample was shipped overnight on dry ice to Metabolon, Inc. (Durham, NC), for broad-spectrum gas and liquid chromatography-mass spectrometry. See Text S1 in the supplemental material for details.

### *In vitro* DC/T-cell fecal water assay.

DCs obtained from anonymous healthy human donors (Blood Centers of the Pacific) were coincubated for 24 h with fecal water prepared from the same fecal samples submitted for metabolite profiling (filter to remove intact cells) prior to stimulation with TNF-α, IL-1β, IL-6, and prostaglandin E2 and incubated for an additional 24 h to induce maturation. DCs were then harvested, washed, and cocultured with autologous T cells at a ratio of 1/10 for 5 days, with medium replenishment every 2 days. The T-cell phenotype was assessed via flow cytometry, and cytokine secretion was assessed by Cytometric Bead Array analysis (BD Biosciences). The assay was repeated in quadruplicate with distinct donors to ensure that observations were not confounded by the peripheral blood mononuclear cell (PBMC) source. For further details, see Text S1 in the supplemental material.

### Statistical analysis. (i) Microbial, metagenomic, and metabolomic analyses.

Statistical analyses were performed with QIIME v1.8.0 and the R statistical environment (47, 49). For PhyloChip data, fluorescence intensities were log normalized prior to analysis. For details of the analyses, see Text S1 in the supplemental material.

### (ii) Comparison of clinical measurements of disease severity.

Clinical measurements of disease severity were compared between UC MCSs by a Kruskal-Wallis test, followed by a pairwise two-tailed Dunn test.

### (iii) Analysis of T-cell subsets.

A linear mixed-effect model was applied with the *lme4* package in R to identify significant differences in the abundance of induced T-cell subpopulations based on sample groups (i.e., UC MCSs) while accounting for potential variation introduced by the PBMC source (i.e., donor) (50). For details, see Text S1 in the supplemental material.

### Microarray and nucleotide sequence data accession numbers.

All microarray data have been deposited in the Gene Expression Omnibus database (http://www.ncbi.nlm.nih.gov/geo) under accession no. GSE78724. All of the sequence data related to this study are available in the Sequence Read Archive database (http://www.ncbi.nlm.nih.gov/sra) under accession no. SRP071201.

## SUPPLEMENTAL MATERIAL

Figure S1  Comparison of healthy (*n =* 13) and UC-associated (*n =* 30) fecal fungal microbiotas. (a) Fungal α diversity stratified by healthy status. (b) Fungal community composition represented by NMDS of pairwise Bray-Curtis distances. Participants are colored by health status. Bacterial α diversity (c) and fungal α diversity (d) were stratified by health status and ethnicity (10 healthy EU, 3 healthy SA, 18 UC EU, 12 UC SA). (e) Simple clinical colitis activity of UC patients stratified by ethnicity (14 EU UC, 12 SA UC). *P* values were obtained by two-tailed rank sum test. (f) Bacterial community composition of all participants stratified by ethnicity (28 EU, 15 SA) represented by NMDS of pairwise weighted UniFrac distances. (g) Fungal community composition of all participants stratified by ethnicity (28 EU, 15 SA) represented by NMDS of pairwise Bray-Curtis distances. (h) PhyloChip-profiled bacterial community composition of UC patients stratified by ethnicity (15 EU UC, 11 SA UC) represented by NMDS of pairwise Canberra distances. In panels a, c, and d, horizontal bars represent means ± standard deviations. *P* values were obtained by two-tailed *t* test. In panels b and f to h, each dashed ellipse represents the 95% confidence interval for the centroid of each participant stratification group as calculated by ordiellipse. Each dot/square represents a single fecal sample obtained from a single donor. Download Figure S1, PDF file, 0.2 MB

Figure S2  Bacterial community compositions of UC patients stratified by UC MCS. (a) NMDS of pairwise weighted UniFrac distances for 16S rRNA profiles obtained via Illumina MiSeq (12 MCS1, 10 MCS2, 4 MCS3, 3 MCS4, 1 other). (b) NMDS of pairwise Canberra distances for 16S rRNA profiles obtained via PhyloChip (10 MCS1, 8 MCS2, 4 MCS3, 2 MCS4, 1 other). Each dashed ellipse represents the 95% confidence interval for the centroid of each participant stratification group as calculated by ordiellipse. Each dot/square represents a single fecal sample obtained from a single donor. (c) Distribution of UC MCSs according to patient ethnicity. The *P* value was obtained by Fisher’s exact test. (d) Bacterial OTUs significantly enriched (red shade) or depleted (blue shade) in MCS1 versus MCS2, -3, or -4, respectively (*P* values, <0.05; see Text S1). Download Figure S2, PDF file, 1 MB

Figure S3  Identification of predominantly EU UC MCSs (MCS1 and -2) in two publicly available data sets (9, 11). Bacterial community composition of UC patients stratified by UC MCS represented by NMDS of pairwise weighted UniFrac distances. (a) Gevers et al., stool samples (*n =* 56). (b) Morgan et al., stool samples (*n =* 47). (c) Gevers et al., biopsy specimens (*n =* 60). (d) Morgan et al., biopsy specimens (*n =* 18). Each dashed ellipse represents the 95% confidence interval for the centroid of each participant stratification group as calculated by ordiellipse. Each dot/square represents a single fecal sample obtained from a single donor. Download Figure S3, PDF file, 0.1 MB

Figure S4  (a) Heat map of KEGG pathways differentially enriched across UC MCSs. The KEGG pathways shown were initially identified by Kruskal-Wallis test comparing distributions among UC MCSs (*q* values, <0.0006). Column order is consistent with Fig. 2. Rows are ordered alphabetically by superpathway, subpathway, and pathway. For visualization, read counts were normalized [log_2_(*x* + 1)] and scaled by row. (b) KEGG pathways significantly enriched (red shade) or depleted (blue shade) in MCS1 versus MCS2, -3, or -4, respectively (*P* values, <0.05; see Text S1). Download Figure S4, PDF file, 0.5 MB

Figure S5  *In vitro* human T-cell activity following coculture with autologous DCs coincubated with sterile fecal water. Induced Th1-to-Th2 ratios of EU UC (*n =*) and SA UC patients are compared. Data were generated from four replicate experiments with DCs/T cells obtained from two anonymous PBMC donors. Horizontal bars (mean fitted values for each group) and *P* values were determined by linear mixed-effect modeling (see Text S1). Download Figure S5, PDF file, 0.02 MB

Table S1  Microbial OTUs differentially enriched among UC patients and healthy participants. (a) Bacterial OTUs that differ significantly between UC patients and healthy controls (MiSeq, *P* value, <0.05; *q* value, <0.3). (b) Bacterial OTUs that differ significantly between UC patients and healthy controls (PhyloChip, Student’s *t* test, *P* value, <0.05; *q* value, <0.3). (c) Fungal OTUs that differ significantly between UC patients and healthy controls (*P* value, <0.05; *q* value, <0.3). Table S1, XLSX file, 0.1 MB

Table S2  Microbial OTUs differentially enriched among UC MCSs. (a) Bacterial OTUs that differ significantly between UC MCSs (MiSeq, Kruskal-Wallis test *P* value, <0.05; *q* values, <0.08). (b) Fungal OTUs that differ significantly between UC MCSs (MiSeq, *P* value, <0.05). Table S2, XLSX file, 0.1 MB

Table S3  KEGG pathways differentially enriched among UC MCSs. (a) KEGG pathways that differ significantly between UC MCSs (PICRUSt, Kruskal-Wallis test, *P* value, <0.05; *q* value, <0.0006). (b) KEGG pathways that differ significantly between MCS1 and MCS2 (PICRUSt, *P* value, <0.05; *q* value, <0.1). Table S3, XLSX file, 0.1 MB

Table S4  Relationship between fecal metabolites and UC MCSs. (a) Mantel test results comparing distance matrices generated from 16S rRNA composition, *in silico* predicted bacterial metagenome, and metabolomic data. (b) Fecal metabolites that differ significantly between MCS1 and MCS2 (Welch’s *t* test, *P* value, <0.05). (c) Fecal metabolites that differ significantly between MCS1 and MCS3 (Welch’s *t* test, *P* value, <0.05). (d) Fecal metabolites that differ significantly between MCS1 and MCS4 (Welch’s *t* test, *P* value, <0.05). Table S4, XLSX file, 0.1 MB

Text S1  Supplemental materials and methods used in this study. Download Text S1, DOCX file, 0.1 MB
