# Supplementary figures and images for: Disease Severity and Immune Activity Relate to Distinct Interkingdom Gut Microbiome States in Ethnically Distinct Ulcerative Colitis Patients
Source: mBio. 2016 Aug 16;7(4):e01072-16. doi: 10.1128/mBio.01072-16 (PMC4992973; doi:10.1128/mBio.01072-16)

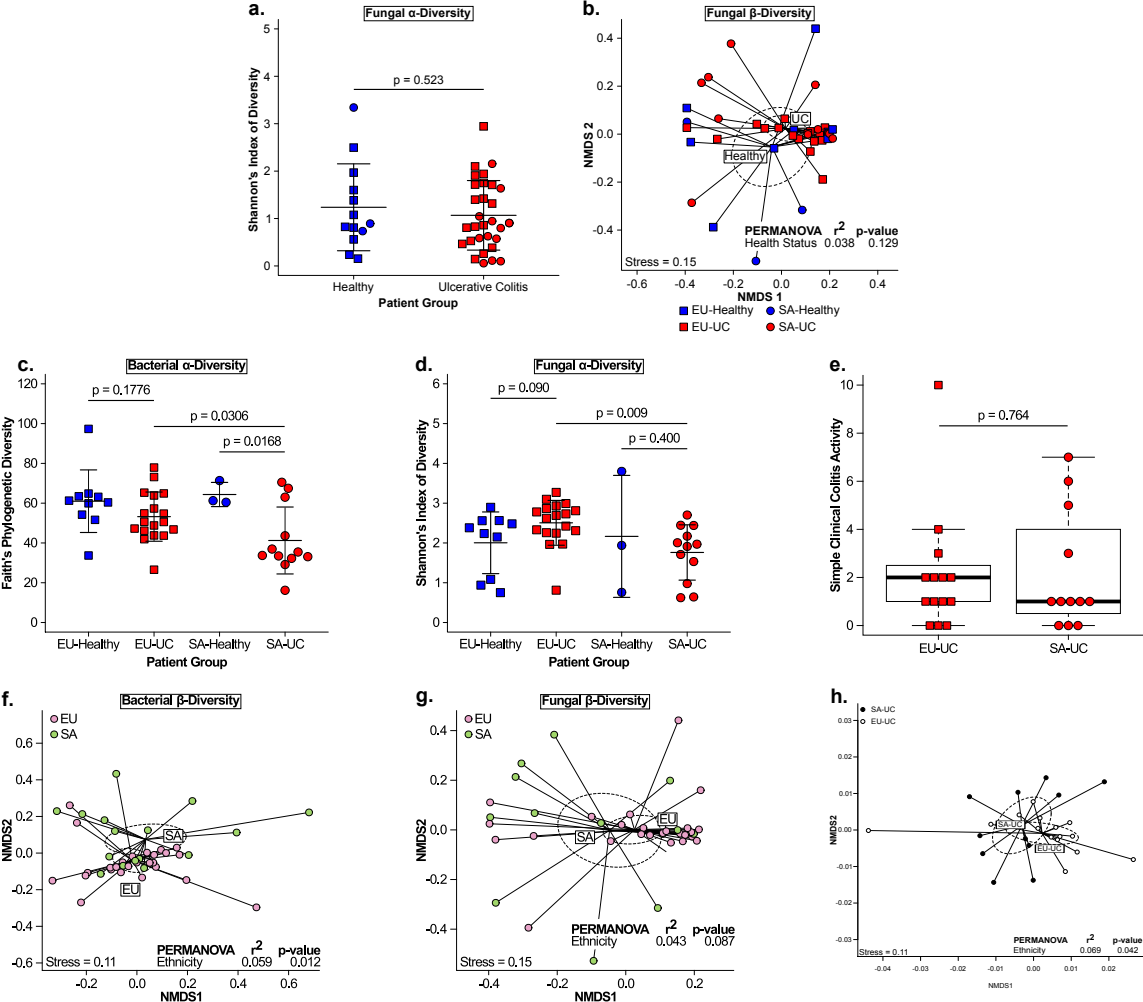

Supplement: Figure S1 — Comparison of healthy (n = 13) and UC-associated (n = 30) fecal fungal microbiotas. (a) Fungal α diversity stratified by healthy status. (b) Fungal community composition represented by NMDS of pairwise Bray-Curtis distances. Participants are colored by health status. Bacterial α diversity (c) and fungal α diversity (d) were stratified by health status and ethnicity (10 healthy EU, 3 healthy SA, 18 UC EU, 12 UC SA). (e) Simple clinical colitis activity of UC patients stratified by ethnicity (14 EU UC, 12 SA UC). P values were obtained by two-tailed rank sum test. (f) Bacterial community composition of all participants stratified by ethnicity (28 EU, 15 SA) represented by NMDS of pairwise weighted UniFrac distances. (g) Fungal community composition of all participants stratified by ethnicity (28 EU, 15 SA) represented by NMDS of pairwise Bray-Curtis distances. (h) PhyloChip-profiled bacterial community composition of UC patients stratified by ethnicity (15 EU UC, 11 SA UC) represented by NMDS of pairwise Canberra distances. In panels a, c, and d, horizontal bars represent means ± standard deviations. P values were obtained by two-tailed t test. In panels b and f to h, each dashed ellipse represents the 95% confidence interval for the centroid of each participant stratification group as calculated by ordiellipse. Each dot/square represents a single fecal sample obtained from a single donor. Download [file mbo004162947sf1.pdf]

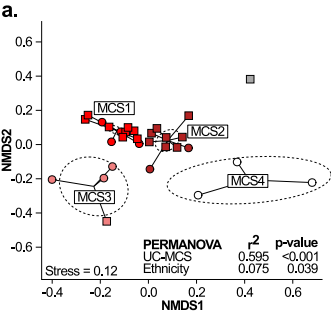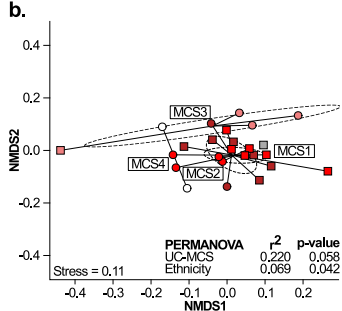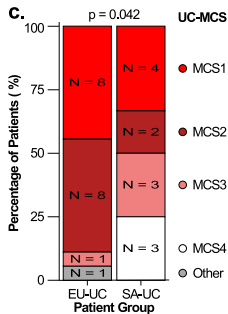

**d.**

## Bacterial Family

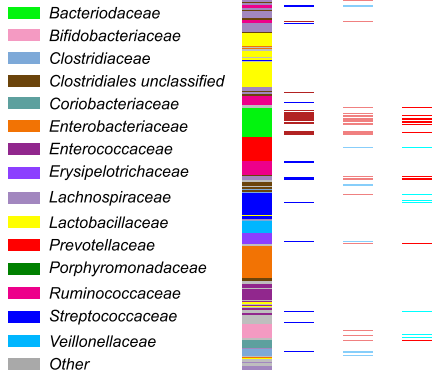

Supplement: Figure S2 — Bacterial community compositions of UC patients stratified by UC MCS. (a) NMDS of pairwise weighted UniFrac distances for 16S rRNA profiles obtained via Illumina MiSeq (12 MCS1, 10 MCS2, 4 MCS3, 3 MCS4, 1 other). (b) NMDS of pairwise Canberra distances for 16S rRNA profiles obtained via PhyloChip (10 MCS1, 8 MCS2, 4 MCS3, 2 MCS4, 1 other). Each dashed ellipse represents the 95% confidence interval for the centroid of each participant stratification group as calculated by ordiellipse. Each dot/square represents a single fecal sample obtained from a single donor. (c) Distribution of UC MCSs according to patient ethnicity. The P value was obtained by Fisher’s exact test. (d) Bacterial OTUs significantly enriched (red shade) or depleted (blue shade) in MCS1 versus MCS2, -3, or -4, respectively (P values, <0.05; see Text S1). Download [file mbo004162947sf2.pdf]

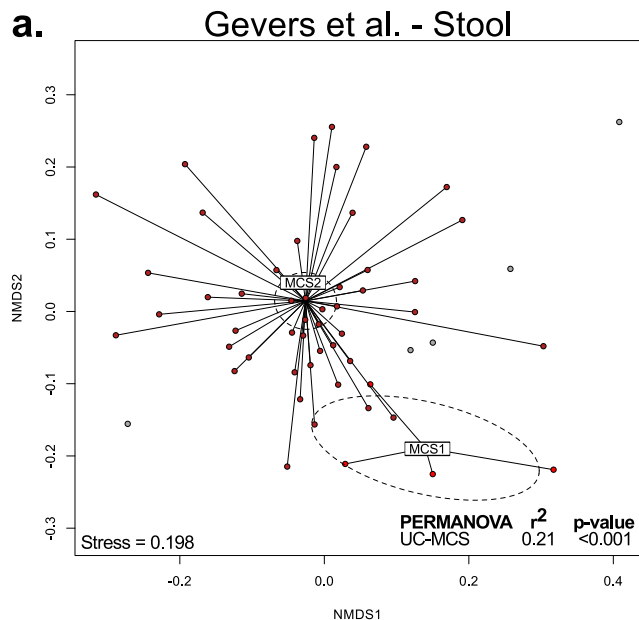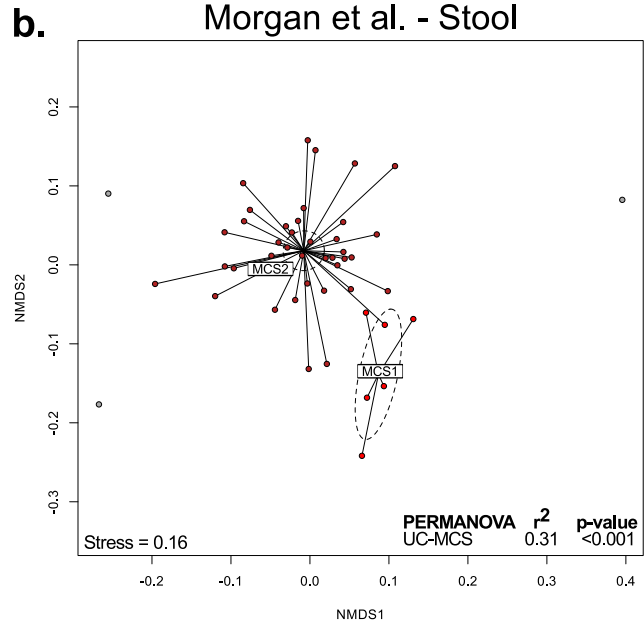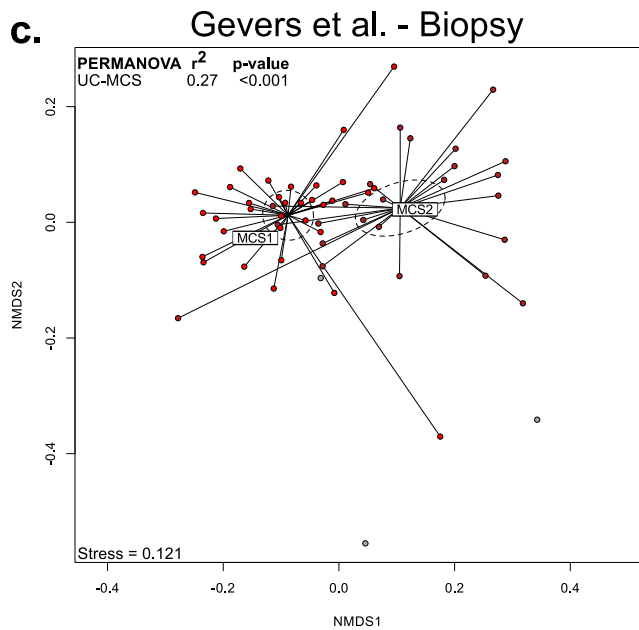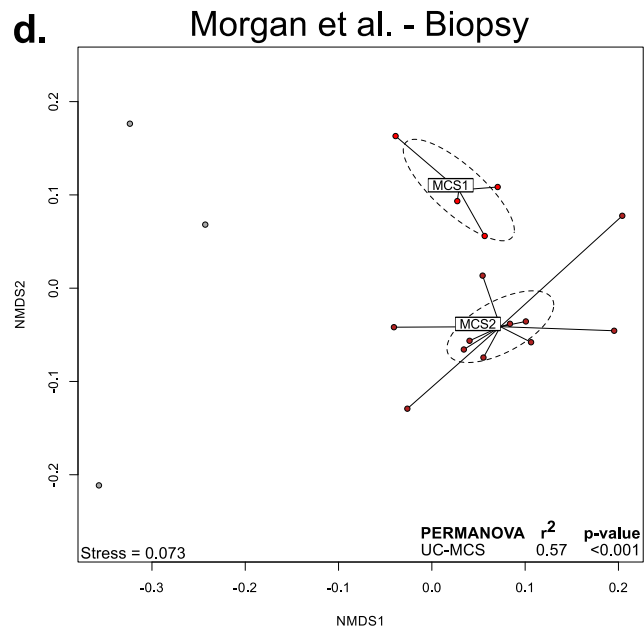

Supplement: Figure S3 — Identification of predominantly EU UC MCSs (MCS1 and -2) in two publicly available data sets (9, 11). Bacterial community composition of UC patients stratified by UC MCS represented by NMDS of pairwise weighted UniFrac distances. (a) Gevers et al., stool samples (n = 56). (b) Morgan et al., stool samples (n = 47). (c) Gevers et al., biopsy specimens (n = 60). (d) Morgan et al., biopsy specimens (n = 18). Each dashed ellipse represents the 95% confidence interval for the centroid of each participant stratification group as calculated by ordiellipse. Each dot/square represents a single fecal sample obtained from a single donor. Download [file mbo004162947sf3.pdf]

**a.**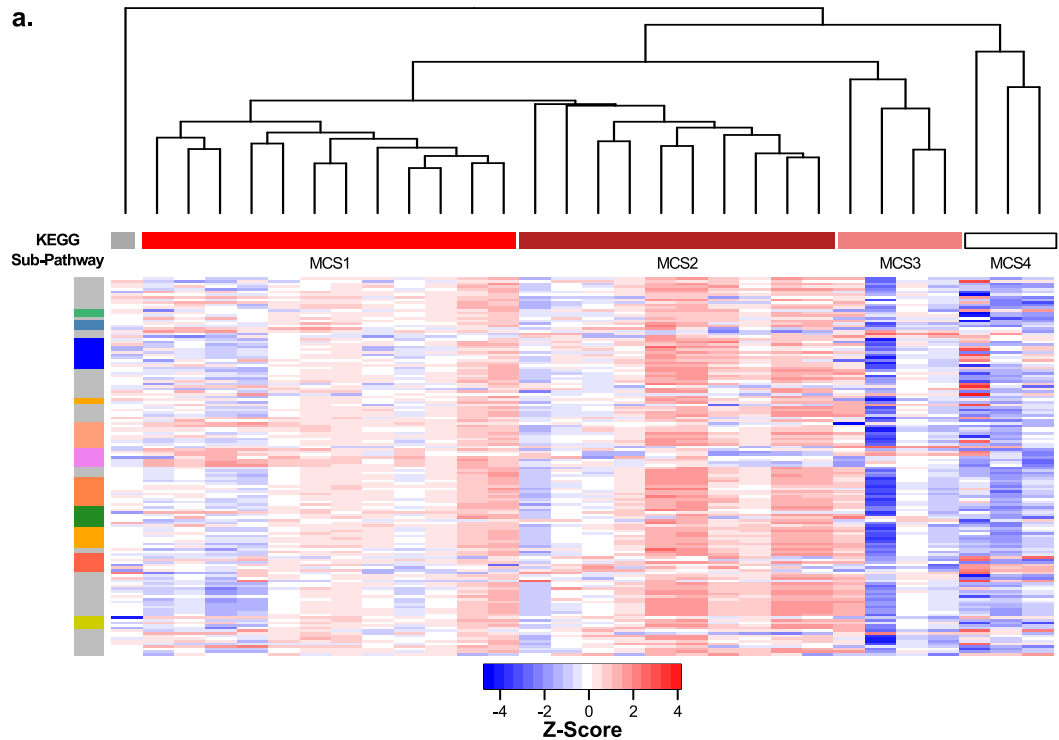**b.**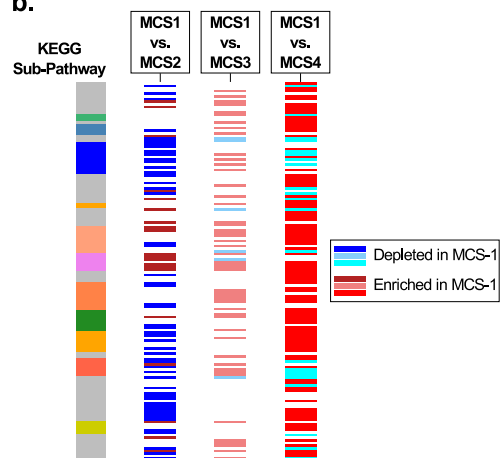

Supplement: Figure S4 — (a) Heat map of KEGG pathways differentially enriched across UC MCSs. The KEGG pathways shown were initially identified by Kruskal-Wallis test comparing distributions among UC MCSs (q values, <0.0006). Column order is consistent with Fig. 2. Rows are ordered alphabetically by superpathway, subpathway, and pathway. For visualization, read counts were normalized [log2(x + 1)] and scaled by row. (b) KEGG pathways significantly enriched (red shade) or depleted (blue shade) in MCS1 versus MCS2, -3, or -4, respectively (P values, <0.05; see Text S1). Download [file mbo004162947sf4.pdf]

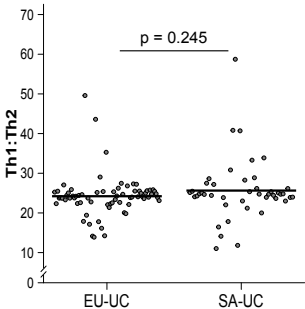

Supplement: Figure S5 — In vitro human T-cell activity following coculture with autologous DCs coincubated with sterile fecal water. Induced Th1-to-Th2 ratios of EU UC (n =) and SA UC patients are compared. Data were generated from four replicate experiments with DCs/T cells obtained from two anonymous PBMC donors. Horizontal bars (mean fitted values for each group) and P values were determined by linear mixed-effect modeling (see Text S1). Download [file mbo004162947sf5.pdf]
