# Supplementary material for: Disease Severity and Immune Activity Relate to Distinct Interkingdom Gut Microbiome States in Ethnically Distinct Ulcerative Colitis Patients
Source: mBio. 2016 Aug 16;7(4):e01072-16. doi: 10.1128/mBio.01072-16 (PMC4992973; doi:10.1128/mBio.01072-16)
Supplement: Text S1 — Supplemental materials and methods used in this study. Download [file mbo004162947s1.docx]

**Supplementary Materials & Methods**

**Fecal Sample Collection**

Study participants were provided detailed instructions and necessary materials for fecal sample collection. Standardized fecal samples (first stool of the morning) were collect at home by defecating onto a sterile stool collection device (Cat. No. Protocult #120; Ability Building Center, MN ) placed over a toilet seat and using a sterile collection cup with an attached sterile scoop (Cat. No. 80.734.311; Sarstedt, Germany). Following collection, fecal samples were placed in a pre-paid overnight mailer with a frozen ice pack (Cat. No. S-9902; ULINE, CA) and shipped overnight via USPS in accordance with federal regulations. Upon arrival, fecal sample were immediately stored at -80ºC. This study was approved by the Committee on Human Research at the University of California, San Francisco (CHR # 10-03092). Physician diagnosed Ulcerative Colitis patients (age 18 to 60 years old) were recruited directly from the gastroenterology clinic at UCSF’s Mount Zion Campus. A questionnaire was provided to each patient to assess clinical measures of disease severity [Simple Clinical Colitis Activity index (SCCA) ([1](#_ENREF_1)), extra-colonic manifestations (arthritis, pyoderma gangrenosum, erythema nodosum, and uveitis), number of first- and second-degree relatives diagnosed with IBD, and duration of disease (years since UC diagnosis)]. Healthy volunteers (age 18 to 60 years old) were drawn from patients’ families and by word of mouth. All participants were self-reported to be of either European or South Asian ethnicity (Table S5). Additionally, all participants resided within a 70-mile radius of San Francisco, CA. Any participant experiencing pregnancy or breast feeding, severe concomitant disease involving the liver, heart, lungs or kidneys, or antibiotic treatment within the preceding 2 months were excluded from the study.

**Table S5.** Breakdown of Study Participant Cohort. Note: one SA-UC participant failed to report their sex.

|  | EU-Healthy | SA-Healthy | EU-UC | SA-UC |
| --- | --- | --- | --- | --- |
| Healthy | 10 | 3 | 18 | 12 |
| Male:Female | 7:3 | 1:2 | 7:11 | 9:2 |
| Median Age (range) | 28.5 (26-67) | 38 (29-59) | 36.5 (22-67) | 43 (24:76) |
| Median BMI (range) | 21.9  (19.94-28.98) | 24.69  (23.06-26.83) | 24.94  (17.47-32.89) | 23.78  (18.47-28.06) |
| Medication History: |  |  |  |  |
| ASA Usage –  Current:Unknown  (MSC-1:-2:-3:-4) | *NA* | *NA* | **9:3**  (5:3:0:NA) | **9:1**  (3:1:3:2) |
| Corticosteroid Usage – Current: Unknown  (MSC-1:-2:-3:-4) | *NA* | *NA* | **4:3**  (2:1:1:NA) | **5:2**  (2:0:1:2) |
| Antimetabolite Usage – Current: Unknown  (MSC-1:-2:-3:-4) | *NA* | *NA* | **7:8**  (4:3:0:NA) | **2:8**  (0:0:1:1) |
| Biologics Usage – Current: Unknown  (MSC-1:-2:-3:-4) | *NA* | *NA* | **5:12**  (3:2:0:NA) | **1:10**  (0:1:0:0) |

**Fecal DNA Isolation**

DNA was extracted from individual fecal samples using a combination of bead beating and the commercially available QIAamp® DNA Stool Kit (Cat. No. 51504; QIAGEN, CA). Initially, 1.6mL of Buffer ASL was added to approximately 100mg of feces and bead beat for 30 s at 6.0 m/s in a FastPrep-24 instrument (Cat. No. 116004500; MP Biomedicals). Following bead beating, samples were incubated at 95ºC for 5 minutes to improve lysis efficiency of difficult to lyse microbes. The remainder of the DNA isolation was conducted using a QIAcube (Cat. No. 9001292; QIAGEN, CA) according to the QIAamp® DNA Stool Kit Protocol: Isolation of DNA from Stool for Pathogen Detection. Isolated DNA was stored at -80ºC. Blank extractions were included as negative controls to monitor for bacterial contamination.

**Bacterial 16S rRNA Gene Library Preparation**

Bacterial 16S rRNA gene sequencing libraries were created as previously described ([2](#_ENREF_2)). PCR amplification of the 16S rRNA gene was conducted in triplicate for each sample using barcoded primers targeting the V4 region (Table S6) as previously described ([2](#_ENREF_2)). Blank extractions were used as template for negative controls to monitor for 16S rRNA contamination. PCR reactions were performed in 25 µl reactions using 0.025 U Takara Hot Start ExTaq (Takara Mirus Bio Inc, Madison, WI), 1X Takara buffer with MgCl_2_, 0.4 pmol µl^-1^ of F515 and R806 primers, 0.56 mg ml^-1^ of bovine serum albumin (BSA; Roche Applied Science, Indianapolis, IN), 200 µM of dNTPs, and 10 ng of gDNA. Reactions were performed in triplicate under the following conditions: initial denaturation (98 °C for 2 min) followed by 30 cycles of 98 °C (20 sec), annealing at 50 °C (30 sec), extension at 72 °C (45 sec) and a final extension at 72 °C for 10 min. Following PCR, triplicates were pooled and 16s rRNA amplicon concentrations were determined via gel electrophoresis quantitation. 16S rRNA sequence library was created by pooling all PCR amplicons in equimolar concentrations to a final volume of 75uL. To remove background, the 16S rRNA sequence library was run on a 2% agarose gel and the 16S amplicon (~380bp) was purified using the QIAquick Gel Extraction Kit (Cat. No. 28704; QIAGEN, CA).

**Table S6.** Primers used for PCR amplication of the 16S rRNA gene

| Sample ID | Golay Barcode Sequence | Linker Sequence | Reverse Primer | Forward Primer |
| --- | --- | --- | --- | --- |
| **1** | TGGTCAACGATA | GTGTGCCAGCMGCCGCGGTAA | GGACTACHVGGGTWTCTAAT | GTGTGCCAGCMGCCGCGGTAA |
| **2** | TCCCTTGTCTCC | GTGTGCCAGCMGCCGCGGTAA | GGACTACHVGGGTWTCTAAT | GTGTGCCAGCMGCCGCGGTAA |
| **3** | GTAGATCGTGTA | GTGTGCCAGCMGCCGCGGTAA | GGACTACHVGGGTWTCTAAT | GTGTGCCAGCMGCCGCGGTAA |
| **4** | TGCATACACTGG | GTGTGCCAGCMGCCGCGGTAA | GGACTACHVGGGTWTCTAAT | GTGTGCCAGCMGCCGCGGTAA |
| **5** | GCGATATATCGC | GTGTGCCAGCMGCCGCGGTAA | GGACTACHVGGGTWTCTAAT | GTGTGCCAGCMGCCGCGGTAA |
| **6** | CGAGGGAAAGTC | GTGTGCCAGCMGCCGCGGTAA | GGACTACHVGGGTWTCTAAT | GTGTGCCAGCMGCCGCGGTAA |
| **9** | CACTACGCTAGA | GTGTGCCAGCMGCCGCGGTAA | GGACTACHVGGGTWTCTAAT | GTGTGCCAGCMGCCGCGGTAA |
| **10** | TACTACGTGGCC | GTGTGCCAGCMGCCGCGGTAA | GGACTACHVGGGTWTCTAAT | GTGTGCCAGCMGCCGCGGTAA |
| **11** | CGGTCAATTGAC | GTGTGCCAGCMGCCGCGGTAA | GGACTACHVGGGTWTCTAAT | GTGTGCCAGCMGCCGCGGTAA |
| **13** | GGTGACTAGTTC | GTGTGCCAGCMGCCGCGGTAA | GGACTACHVGGGTWTCTAAT | GTGTGCCAGCMGCCGCGGTAA |
| **14** | CGTAAGATGCCT | GTGTGCCAGCMGCCGCGGTAA | GGACTACHVGGGTWTCTAAT | GTGTGCCAGCMGCCGCGGTAA |
| **15** | ACGAGACTGATT | GTGTGCCAGCMGCCGCGGTAA | GGACTACHVGGGTWTCTAAT | GTGTGCCAGCMGCCGCGGTAA |
| **16** | AGTCGAACGAGG | GTGTGCCAGCMGCCGCGGTAA | GGACTACHVGGGTWTCTAAT | GTGTGCCAGCMGCCGCGGTAA |
| **17** | CGAGCAATCCTA | GTGTGCCAGCMGCCGCGGTAA | GGACTACHVGGGTWTCTAAT | GTGTGCCAGCMGCCGCGGTAA |
| **22** | TGCAGTCCTCGA | GTGTGCCAGCMGCCGCGGTAA | GGACTACHVGGGTWTCTAAT | GTGTGCCAGCMGCCGCGGTAA |
| **24** | GGCCAGTTCCTA | GTGTGCCAGCMGCCGCGGTAA | GGACTACHVGGGTWTCTAAT | GTGTGCCAGCMGCCGCGGTAA |
| **25** | GTGGAGTCTCAT | GTGTGCCAGCMGCCGCGGTAA | GGACTACHVGGGTWTCTAAT | GTGTGCCAGCMGCCGCGGTAA |
| **26** | ATGGGTTCCGTC | GTGTGCCAGCMGCCGCGGTAA | GGACTACHVGGGTWTCTAAT | GTGTGCCAGCMGCCGCGGTAA |
| **27** | GCGTTCTAGCTG | GTGTGCCAGCMGCCGCGGTAA | GGACTACHVGGGTWTCTAAT | GTGTGCCAGCMGCCGCGGTAA |
| **28** | GCTGTACGGATT | GTGTGCCAGCMGCCGCGGTAA | GGACTACHVGGGTWTCTAAT | GTGTGCCAGCMGCCGCGGTAA |
| **29** | ACCAGTGACTCA | GTGTGCCAGCMGCCGCGGTAA | GGACTACHVGGGTWTCTAAT | GTGTGCCAGCMGCCGCGGTAA |
| **31** | AGTCGTGCACAT | GTGTGCCAGCMGCCGCGGTAA | GGACTACHVGGGTWTCTAAT | GTGTGCCAGCMGCCGCGGTAA |
| **32** | ACCATAGCTCCG | GTGTGCCAGCMGCCGCGGTAA | GGACTACHVGGGTWTCTAAT | GTGTGCCAGCMGCCGCGGTAA |
| **33** | GATGTTCGCTAG | GTGTGCCAGCMGCCGCGGTAA | GGACTACHVGGGTWTCTAAT | GTGTGCCAGCMGCCGCGGTAA |
| **34** | GCTCGAAGATTC | GTGTGCCAGCMGCCGCGGTAA | GGACTACHVGGGTWTCTAAT | GTGTGCCAGCMGCCGCGGTAA |
| **37** | TAGGCATGCTTG | GTGTGCCAGCMGCCGCGGTAA | GGACTACHVGGGTWTCTAAT | GTGTGCCAGCMGCCGCGGTAA |
| **38** | GTTGTTCTGGGA | GTGTGCCAGCMGCCGCGGTAA | GGACTACHVGGGTWTCTAAT | GTGTGCCAGCMGCCGCGGTAA |
| **40** | ATCACCAGGTGT | GTGTGCCAGCMGCCGCGGTAA | GGACTACHVGGGTWTCTAAT | GTGTGCCAGCMGCCGCGGTAA |
| **41** | GAATACCAAGTC | GTGTGCCAGCMGCCGCGGTAA | GGACTACHVGGGTWTCTAAT | GTGTGCCAGCMGCCGCGGTAA |
| **43** | GTATCTGCGCGT | GTGTGCCAGCMGCCGCGGTAA | GGACTACHVGGGTWTCTAAT | GTGTGCCAGCMGCCGCGGTAA |
| **44** | TCGACATCTCTT | GTGTGCCAGCMGCCGCGGTAA | GGACTACHVGGGTWTCTAAT | GTGTGCCAGCMGCCGCGGTAA |
| **46** | CTATCTCCTGTC | GTGTGCCAGCMGCCGCGGTAA | GGACTACHVGGGTWTCTAAT | GTGTGCCAGCMGCCGCGGTAA |
| **49** | AGGCTTACGTGT | GTGTGCCAGCMGCCGCGGTAA | GGACTACHVGGGTWTCTAAT | GTGTGCCAGCMGCCGCGGTAA |
| **50** | AACTAGTTCAGG | GTGTGCCAGCMGCCGCGGTAA | GGACTACHVGGGTWTCTAAT | GTGTGCCAGCMGCCGCGGTAA |
| **51** | GGACTTCCAGCT | GTGTGCCAGCMGCCGCGGTAA | GGACTACHVGGGTWTCTAAT | GTGTGCCAGCMGCCGCGGTAA |
| **54** | GAACACTTTGGA | GTGTGCCAGCMGCCGCGGTAA | GGACTACHVGGGTWTCTAAT | GTGTGCCAGCMGCCGCGGTAA |
| **55** | ACTCACAGGAAT | GTGTGCCAGCMGCCGCGGTAA | GGACTACHVGGGTWTCTAAT | GTGTGCCAGCMGCCGCGGTAA |
| **58** | TAACGTGTGTGC | GTGTGCCAGCMGCCGCGGTAA | GGACTACHVGGGTWTCTAAT | GTGTGCCAGCMGCCGCGGTAA |
| **59** | TCTCTACCACTC | GTGTGCCAGCMGCCGCGGTAA | GGACTACHVGGGTWTCTAAT | GTGTGCCAGCMGCCGCGGTAA |
| **74** | ATTCTGCCGAAG | GTGTGCCAGCMGCCGCGGTAA | GGACTACHVGGGTWTCTAAT | GTGTGCCAGCMGCCGCGGTAA |
| **75** | CAAATTCGGGAT | GTGTGCCAGCMGCCGCGGTAA | GGACTACHVGGGTWTCTAAT | GTGTGCCAGCMGCCGCGGTAA |
| **78** | CTCACAACCGTG | GTGTGCCAGCMGCCGCGGTAA | GGACTACHVGGGTWTCTAAT | GTGTGCCAGCMGCCGCGGTAA |
| **79** | ATCGCACAGTAA | GTGTGCCAGCMGCCGCGGTAA | GGACTACHVGGGTWTCTAAT | GTGTGCCAGCMGCCGCGGTAA |

**Fungal ITS2 Library Preparation**

Fungal internal transcribed spacer 2 (ITS2) sequencing libraries were created using similar methods to those used for the 16S rRNA library. PCR amplification of the ITS2 region was conducted in triplicate for each sample using barcoded primers (Table S7). PCR reactions were performed in 25 µl reaction with 1X Takara buffer (Takara Mirus Bio), 200 nM of each primer, 200 µM dNTPs, 2.75 mM of MgCl_2_, 0.56 mg ml^-1^ of BSA (Roche Applied Science), 0.025 U Takara Hot Start ExTaq and 50 ng of gDNA. Reactions were conducted under the following conditions: initial denaturation (94 °C for 5 min) followed by 30 cycles of 94 °C (30 sec), annealing at 54 °C (30 sec), extension at 72 °C (30 sec) and a final extension at 72 °C for 7 min. Following PCR, triplicates were pooled and purified using the Agencourt AMPure XP - PCR Purification Kit and associated protocol (Cat. No. A63880, Beckman Coulter). Samples were quantified using the KAPA SYBR FAST qPCR Kit (Cat. No. KK4601, KAPA Biosystems) as recommended by the manufacturers. All purified samples were then pooled in equimolar concentrations based individual sample ITS2 quantification to a final volume of 75uL.

**Supplemental Table S7:** Primers used for PCR amplication of the ITS2 region

| Sample ID | Golay Barcode Sequence | Linker Sequence | Reverse Primer | Forward Primer |
| --- | --- | --- | --- | --- |
| **1** | GTAATGCGTAAC | GTGTGCCAGCMGCCGCGGTAA | TCCTCCGCTTATTGATATGC | GTGARTCATCGAATCTTTG |
| **2** | GTCGAATTTGCG | GTGTGCCAGCMGCCGCGGTAA | TCCTCCGCTTATTGATATGC | GTGARTCATCGAATCTTTG |
| **3** | GTCGCCGTACAT | GTGTGCCAGCMGCCGCGGTAA | TCCTCCGCTTATTGATATGC | GTGARTCATCGAATCTTTG |
| **4** | GCATCAGAGTTA | GTGTGCCAGCMGCCGCGGTAA | TCCTCCGCTTATTGATATGC | GTGARTCATCGAATCTTTG |
| **5** | GTGGTCATCGTA | GTGTGCCAGCMGCCGCGGTAA | TCCTCCGCTTATTGATATGC | GTGARTCATCGAATCTTTG |
| **6** | GGAATCCGATTA | GTGTGCCAGCMGCCGCGGTAA | TCCTCCGCTTATTGATATGC | GTGARTCATCGAATCTTTG |
| **9** | CTGAAGGGCGAA | GTGTGCCAGCMGCCGCGGTAA | TCCTCCGCTTATTGATATGC | GTGARTCATCGAATCTTTG |
| **10** | CGCTCACAGAAT | GTGTGCCAGCMGCCGCGGTAA | TCCTCCGCTTATTGATATGC | GTGARTCATCGAATCTTTG |
| **11** | ATTCGGTAGTGC | GTGTGCCAGCMGCCGCGGTAA | TCCTCCGCTTATTGATATGC | GTGARTCATCGAATCTTTG |
| **13** | CGAGCTGTTACC | GTGTGCCAGCMGCCGCGGTAA | TCCTCCGCTTATTGATATGC | GTGARTCATCGAATCTTTG |
| **14** | CAACACATGCTG | GTGTGCCAGCMGCCGCGGTAA | TCCTCCGCTTATTGATATGC | GTGARTCATCGAATCTTTG |
| **15** | ATTCTCTCACGT | GTGTGCCAGCMGCCGCGGTAA | TCCTCCGCTTATTGATATGC | GTGARTCATCGAATCTTTG |
| **16** | CGACTCTAAACG | GTGTGCCAGCMGCCGCGGTAA | TCCTCCGCTTATTGATATGC | GTGARTCATCGAATCTTTG |
| **17** | GTCTTCAGCAAG | GTGTGCCAGCMGCCGCGGTAA | TCCTCCGCTTATTGATATGC | GTGARTCATCGAATCTTTG |
| **22** | GAACGGGACGTA | GTGTGCCAGCMGCCGCGGTAA | TCCTCCGCTTATTGATATGC | GTGARTCATCGAATCTTTG |
| **24** | ACGTGTAGGCTT | GTGTGCCAGCMGCCGCGGTAA | TCCTCCGCTTATTGATATGC | GTGARTCATCGAATCTTTG |
| **25** | TACGGATTATGG | GTGTGCCAGCMGCCGCGGTAA | TCCTCCGCTTATTGATATGC | GTGARTCATCGAATCTTTG |
| **26** | GCCTGTCTGCAA | GTGTGCCAGCMGCCGCGGTAA | TCCTCCGCTTATTGATATGC | GTGARTCATCGAATCTTTG |
| **27** | ATAGCGAACTCA | GTGTGCCAGCMGCCGCGGTAA | TCCTCCGCTTATTGATATGC | GTGARTCATCGAATCTTTG |
| **28** | TTCGATGCCGCA | GTGTGCCAGCMGCCGCGGTAA | TCCTCCGCTTATTGATATGC | GTGARTCATCGAATCTTTG |
| **29** | CCAGATATAGCA | GTGTGCCAGCMGCCGCGGTAA | TCCTCCGCTTATTGATATGC | GTGARTCATCGAATCTTTG |
| **31** | AACTTTCAGGAG | GTGTGCCAGCMGCCGCGGTAA | TCCTCCGCTTATTGATATGC | GTGARTCATCGAATCTTTG |
| **32** | TAACGCTGTGTG | GTGTGCCAGCMGCCGCGGTAA | TCCTCCGCTTATTGATATGC | GTGARTCATCGAATCTTTG |
| **33** | AACCAAACTCGA | GTGTGCCAGCMGCCGCGGTAA | TCCTCCGCTTATTGATATGC | GTGARTCATCGAATCTTTG |
| **34** | GGTCTCCTACAG | GTGTGCCAGCMGCCGCGGTAA | TCCTCCGCTTATTGATATGC | GTGARTCATCGAATCTTTG |
| **37** | GAGAGTCCACTT | GTGTGCCAGCMGCCGCGGTAA | TCCTCCGCTTATTGATATGC | GTGARTCATCGAATCTTTG |
| **38** | TTCTCCATCACA | GTGTGCCAGCMGCCGCGGTAA | TCCTCCGCTTATTGATATGC | GTGARTCATCGAATCTTTG |
| **40** | CTGGGTATCTCG | GTGTGCCAGCMGCCGCGGTAA | TCCTCCGCTTATTGATATGC | GTGARTCATCGAATCTTTG |
| **41** | GACTACCCGTTG | GTGTGCCAGCMGCCGCGGTAA | TCCTCCGCTTATTGATATGC | GTGARTCATCGAATCTTTG |
| **43** | ATGGGCGAATGG | GTGTGCCAGCMGCCGCGGTAA | TCCTCCGCTTATTGATATGC | GTGARTCATCGAATCTTTG |
| **44** | GATCTCTGGGTA | GTGTGCCAGCMGCCGCGGTAA | TCCTCCGCTTATTGATATGC | GTGARTCATCGAATCTTTG |
| **46** | CATCATACGGGT | GTGTGCCAGCMGCCGCGGTAA | TCCTCCGCTTATTGATATGC | GTGARTCATCGAATCTTTG |
| **49** | CAACGTGCTCCA | GTGTGCCAGCMGCCGCGGTAA | TCCTCCGCTTATTGATATGC | GTGARTCATCGAATCTTTG |
| **50** | TACACAAGTCGC | GTGTGCCAGCMGCCGCGGTAA | TCCTCCGCTTATTGATATGC | GTGARTCATCGAATCTTTG |
| **51** | GCGTCCATGAAT | GTGTGCCAGCMGCCGCGGTAA | TCCTCCGCTTATTGATATGC | GTGARTCATCGAATCTTTG |
| **54** | CACCCGATGGTT | GTGTGCCAGCMGCCGCGGTAA | TCCTCCGCTTATTGATATGC | GTGARTCATCGAATCTTTG |
| **55** | AATGACCTCGTG | GTGTGCCAGCMGCCGCGGTAA | TCCTCCGCTTATTGATATGC | GTGARTCATCGAATCTTTG |
| **58** | GCGTTGCAAACT | GTGTGCCAGCMGCCGCGGTAA | TCCTCCGCTTATTGATATGC | GTGARTCATCGAATCTTTG |
| **59** | ATCCCTACGGAA | GTGTGCCAGCMGCCGCGGTAA | TCCTCCGCTTATTGATATGC | GTGARTCATCGAATCTTTG |
| **74** | GGTTCCATTAGG | GTGTGCCAGCMGCCGCGGTAA | TCCTCCGCTTATTGATATGC | GTGARTCATCGAATCTTTG |
| **75** | TTCCTAGGCCAG | GTGTGCCAGCMGCCGCGGTAA | TCCTCCGCTTATTGATATGC | GTGARTCATCGAATCTTTG |
| **78** | ACCTTACACCTT | GTGTGCCAGCMGCCGCGGTAA | TCCTCCGCTTATTGATATGC | GTGARTCATCGAATCTTTG |
| **79** | CCGAGGTATAAT | GTGTGCCAGCMGCCGCGGTAA | TCCTCCGCTTATTGATATGC | GTGARTCATCGAATCTTTG |

**16S and ITS2 Library Sequencing**

Purified sequencing libraries were analyzed using a Bioanalyzer (Aligent), quantified using the Qubit HS dsDNA kit (Invitrogen), and diluted to 2 nM. Diluted sequence libraries were then denatured, diluted to 5.88pM, and combined with denatured 12.5pM PhiX spike-in to final concentration of 5pM. Prepared sequencing libraries were then loaded onto the Illumina MiSeq cartridge (Cat. No. MS-102-3001, Illumina) and sequenced (514 cycles, Read 1: 251 cycles, Index Read: 12 cycles, Read 2: 251 cycles) using a MiSeq platform and MiSeq Control Software v2.2.0 according to the manufacturer’s instructions (Illumina). All sequence data related to this study is available in the Sequence Read Archive (SRA) database, <http://www.ncbi.nlm.nih.gov/sra> (accession no. PRJNA313074).

*Bacterial 16S rRNA Sequence Processing.* Following paired-end sequencing, paired sequences were assembled using FLASH v1.2.7 with a minimum overlap set at 15bp ([3](#_ENREF_3)). Assembled reads were de-multiplexed by barcode and filtered for low quality (Q-score < 30) using QIIME 1.8 ([4](#_ENREF_4)). If the Q-score three consecutive bases were <30, the read was truncated before the low-quality bases. The resulting read was retained in the dataset if it was at least 75% of the original length. Operational taxonomic units (OTUs) were picked at 97% sequence identity using uclust against the GreenGenes 13_8 database ([5](#_ENREF_5)) ([6](#_ENREF_6)), retaining OTUs containing >1 sequence read. Reads that failed to hit the reference sequence collection were retained and clustered *de novo*. Sequences were aligned using PyNAST and taxonomy was assigned using uclust and the GreenGenes 13_8 database ([7](#_ENREF_7)) ([5](#_ENREF_5)) ([6](#_ENREF_6)). PyNAST-aligned sequences were chimera checked using ChimeraSlayer ([8](#_ENREF_8)), removing putative chimeras and representative sequences that failed PyNAST alignment. A phylogenetic tree was built using FastTree ([9](#_ENREF_9)). To normalize variation in read depth across samples, data were rarefied to the minimum read depth of 49,518 sequences per sample for bacteria. To ensure that a truly representative community of each sample was used for analysis, sequence sub-sampling at the defined depth was bootstrapped 100 times. The representative community composition for each sample was defined as that which exhibited the minimum average Canberra distance to all other OTU vectors generated from all sub-samplings for that particular sample.

*Fungal ITS2 Sequence Processing.* Following paired-end sequencing, paired sequences were assembled using FLASH v1.2.7 with a minimum overlap of 25 bp and a maximum overlap of 290bp ([3](#_ENREF_3)). Assembled reads were de-multiplexed by barcode using QIIME 1.8 ([4](#_ENREF_4)). Assembled reads containing >2 expected errors, as determined by usearch ([5](#_ENREF_5)), were removed. Singleton reads were removed and OTUs of 97% sequence similarity were generated *de novo* using usearch8.0 ([5](#_ENREF_5)). The 8_1_2015 UNITE ITS fungal sequence database and usearch8.0 was used to remove potentially chimeric sequences ([10](#_ENREF_10)) ([5](#_ENREF_5)). The ITSx software package was then used to extract the predicted ITS2 region from the reference sequence of non-chimeric OTUs, filtering out OTUs predicted to lack a true ITS2 region in the process ([11](#_ENREF_11)). Taxonomy was then assigned to non-chimeric, ITS2 extracted OTUs using Bayesian classification with a confidence cut-off of 0.8 in QIIME according to the 8_1_2015 UNITE ITS fungal sequence database ([4](#_ENREF_4)) ([10](#_ENREF_10)). OTUs responsible for less that 0.001% of the total sequence reads were removed. To normalize variation in read depth across samples, data were rarefied to the minimum read depth of 6,653 sequences per sample for bacteria. To ensure that a truly representative community of each sample was used for analysis, sequence sub-sampling at the defined depth was bootstrapped 100 times. The representative community composition for each sample was defined as that which exhibited the minimum average Canberra distance to all other OTU vectors generated from all sub-samplings for that particular sample.

**Bacterial 16S rRNA Gene Profiling Using PhyloChip**

Total DNA extracted from fecal samples was used as template for 16S rRNA gene amplification as previously described ([12](#_ENREF_12)). PCR amplification was verified on a 1% TBE agarose gel then purified using the QIAquick Gel Extraction kit (Cat. No. 28704; QIAGEN, CA). A total of 500ng of purified PCR product per sample was then fragmented, biotin-labeled, and hybridized to the G3 16S rRNA PhyloChip (Affymetrix, CA) as previously described ([13](#_ENREF_13)). Washing, staining, and scanning of arrays were conducted according to standard Affymetrix protocol ([13](#_ENREF_13)). Background subtraction, detection, taxon quantification criteria and array normalization was performed as previously described ([13](#_ENREF_13)). Stage 1 thresholds were adjusted, based on quantitative standards to the following: rQ1 ≥ 0.25, rQ2 ≥ 0.50, rQ3 ≥ 0.80. All PhyloChip microarray data reported in this paper has been deposited in the Gene Expression Omnibus (GEO) database, [www.ncbi.nlm.nih.gov/geo](http://www.ncbi.nlm.nih.gov/geo) (accession no. GSE78724).

**Predicted community metagenome analyses**

Phylogenetic Investigation of Communities by Reconstruction of Unobserved States (PICRUSt; <http://picrust.github.io/picrust/>), a bioinformatics software package used to predict functional metagenomes from a marker gene survey (such as 16S rRNA gene), was used to generate *in silico* bacterial metagenomes for data generated in this study ([14](#_ENREF_14)). First, the biom-formatted bacterial OTU table previously generated from the processed 16S rRNA gene MiSeq data was filtered to contain only closed-reference OTUs [i.e. OTUs present in the GreenGenes 16S rRNA 13_8 database ([6](#_ENREF_6))]. The closed-reference OTU table was then used to generate predicted metagenomes according to the PICRUSt metagenome prediction tutorial ([http://picrust.github.io/picrust/tutorials/metagenome_prediction.html - metagenome-prediction-tutorial](http://picrust.github.io/picrust/tutorials/metagenome_prediction.html#metagenome-prediction-tutorial)). Briefly, OTU abundance was first normalized according to known or predicted 16s copy number. Following 16s copy number normalization, this normalized OTU table was then used to predicted KEGG Ortholog (KO) abundances for each sample, which were further collapsed into KEGG Pathways (<http://www.genome.jp/kegg/pathway.html>).

**Metabolome Profiling**

To profile fecal metabolites, >200mg of frozen stool from each sample was shipped overnight on dry ice to Metabolon (Metabolon, NC). Also included were several technical replicate samples created from a homogeneous pool containing a small amount of all study sample. Upon receipt, samples were inventoried, and immediately stored at -80ºC. At the time of analysis, samples were extracted and prepared for analysis using Metabolon’s standard solvent extraction method (<http://www.metabolon.com/>). The extracted samples were split into equal parts for analysis on the GC/MS and Q-Exactive accurate mass LC/MS platforms.

*Sample Preparation:* The sample preparation process was carried out using the automated MicroLab STAR® system from Hamilton Company. Recovery standards were added prior to the first step in the extraction process for QC purposes. Sample preparation was conducted using a proprietary series of organic and aqueous extractions to remove the protein fraction while allowing maximum recovery of small molecules. The resulting extract was divided into two fractions; one for analysis by LC/MS and one for analysis by GC/MS. Samples were placed briefly on a TurboVap® (Zymark) to remove the organic solvent. Each sample was then frozen and dried under vacuum. Samples were then prepared for the appropriate instrument, either LC/MS or GC/MS.

*QA/QC:* For QA/QC purposes, a number of additional samples were included with each day’s analysis. Furthermore, a selection of QC compounds was added to every sample, including those under test. These compounds were chosen so as not to interfere with the measurement of the endogenous compounds. Tables S8 and S9 describe the QC samples and compounds. These QC samples are primarily used to evaluate the process control for each study as well as aiding in the data curation.

**Table S8:** Description of Metabolon QC Samples

| **Type** | **Description** | **Purpose** |
| --- | --- | --- |
| MTRX | Large pool of human plasma maintained by Metabolon that has been characterized extensively. | Assure that all aspects of Metabolon process are operating within specifications. |
| CMTRX | Pool created by taking a small aliquot from every customer sample. | Assess the effect of a non-plasma matrix on the Metabolon process and distinguish biological variability from process variability. |
| PRCS | Aliquot of ultra-pure water | Process Blank used to assess the contribution to compound signals from the process. |
| SOLV | Aliquot of solvents used in extraction. | Solvent blank used to segregate contamination sources in the extraction. |

**Table S9:** Metabolon QC Standards

| **Type** | **Description** | **Purpose** |
| --- | --- | --- |
| DS | Derivatization Standard | Assess variability of derivatization for GC/MS samples. |
| IS | Internal Standard | Assess variability and performance of instrument. |
| RS | Recovery Standard | Assess variability and verify performance of extraction and instrumentation. |

*Ultrahigh Performance Liquid Chromatography/Mass Spectroscopy (UPLC/MS/MS):* The LC/MS portion of the platform was based on a Waters ACQUITY ultra-performance liquid chromatography (UPLC) and a Thermo Scientific Q-Exactive high resolution/accurate mass spectrometer interfaced with a heated electrospray ionization (HESI-II) source and Orbitrap mass analyzer operated at 35,000 mass resolution. The sample extract was dried then reconstituted in acidic or basic LC-compatible solvents, each of which contained 8 or more injection standards at fixed concentrations to ensure injection and chromatographic consistency. One aliquot was analyzed using acidic positive ion optimized conditions and the other using basic negative ion optimized conditions in two independent injections using separate dedicated columns (Waters UPLC BEH C18-2.1×100 mm, 1.7 µm). Extracts reconstituted in acidic conditions were gradient eluted using water and methanol containing 0.1% formic acid, while the basic extracts, which also used water/methanol, contained 6.5mM Ammonium Bicarbonate. The MS analysis alternated between MS and data-dependent MS2 scans using dynamic exclusion, and the scan range was from 80-1000 m/z. Raw data files are archived and extracted as described below.

*Gas chromatography/Mass Spectrometry (GC/MS):* The samples destined for GC/MS analysis were re-dried under vacuum desiccation for a minimum of 24 hours prior to being derivatized under dried nitrogen using bistrimethyl-silyl-triflouroacetamide (BSTFA). The GC column was 5% phenyl/ 95% dimethyl polysiloxane fused silica column and the temperature ramp was from 40° to 300° C in a 16 minute period. Samples were analyzed on a Thermo-Finnigan Trace DSQ fast-scanning single-quadrupole mass spectrometer using electron impact ionization. The instrument was tuned and calibrated for mass resolution and mass accuracy on a daily basis. The information output from the raw data files was automatically extracted as discussed below.

*Data Extraction and Compound Identification:* Raw data was extracted, peak-identified and QC processed using Metabolon’s hardware and software. Compounds were identified by comparison to library entries of purified standards or recurrent unknown entities. Metabolon maintains a library based on authenticated standards containing the retention time/index (RI), mass to charge ratio (m/z), and chromatographic data (including MS/MS spectral data) on all molecules present in the library. Furthermore, biochemical identifications are based on three criteria: retention index within a narrow RI window of the proposed identification, nominal mass match to the library +/- 0.4 amu, and the MS/MS forward and reverse scores between the experimental data and authentic standards. The MS/MS scores are based on a comparison of the ions present in the experimental spectrum to the ions present in the library spectrum.

*Normalization:* For studies spanning multiple days, a data normalization step was performed to correct variation resulting from instrument inter-day tuning differences. Essentially, each compound was corrected in run-day blocks by registering the medians to equal one (1.00) and normalizing each data point proportionately. For studies that did not require more than one day of analysis, no normalization was necessary.

***In vitro* DC/T-cell fecal water assay**

*Fecal Water Preparation.* Fecal samples were diluted in sterile 37°C PBS containing 20% FBS and 2mM EDTA to a final concentration of 1g/mL. Diluted fecal samples were then vortex for 1 minutes and incubated at 37°C for 10 minutes. Following incubation, samples were centrifuged at ~21,000g for 10 minutes at room temperature to remove insoluble material. Supernatants were then filtered through a 0.2μm nylon filter to remove intact cells. Sterile fecal water solutions were stored at -20ºC.

*Dendritic cell fecal water challenge and T-cell co-culture.* Peripheral blood samples were obtained from anonymous healthy human donors (Blood Centers of the Pacific, San Francisco, CA). Peripheral blood mononuclear cells (PBMCs) were isolated by Ficoll-Hypaque gradient centrifugation (Cat. No. Histopaque-10771; Sigma-Aldrich). Dendritic cells (DCs) were purified from isolated PBMCs using the EasySep™ Human Pan-DC Pre-Enrichment Kit (Cat. No. 19251; STEMCELL Technologies, Canada) and cultured in 96-well plates (0.5 x 10^6^ cells/ml) in fresh R10 media: RPMI 1640 (Cat. No. 11875; Thermo-Fisher Scientific) supplemented with 10% heat-inactivated FCS (Cat. No. 9871-5244; USA Scientific), 100 U/ml penicillin-streptomycin (Cat. No. 10378016; Life Technologies, CA), 10 ng/ml GM-CSF (Cat. No. 15-GM-010; R&D Systems, MN), and 20 ng/ml IL-4 (Cat. No. 204-IL-010; R&D Systems). Prepared sterile fecal water was added to DC culture at a 1/20 dilution. After a 24 hour incubation, cells were stimulated with 10 ng/ml TNF-α (Cat. No. 300-01A; PeproTech, NJ), 10 ng/ml IL-1β (Cat. No. 200-01B; PeproTech), 10 ng/ml IL-6 (Cat. No. AF-200-06; PeproTech), and 1μM PGE2 (Cat. No. 72194; STEMCELL Technologies) and incubated for an additional 24 hours to induce DC maturation. T-cells were purified from autologous, monocyte-depleted PBMCs by negative selection using the Human T-Cell Enrichment Column (Cat. No. HTCC-2000; R&D Systems) and were subsequently cultured in TexMACS Medium (Cat. Not. 130-097-196; Miltenyi Biotec, Germany). Following DC stimulation, DCs were harvested, washed, and co-cultured with autologous T-cells at a ratio of 1/10 in the presence of 1ug/ml soluble anti-CD28 (Cat. No. 555725; BD Biosciences, CA) and 1 μg/ml anti-CD49d (Cat. No. 555501; BD Biosciences) for 5 days, replenishing the media every 2 days. This assay was repeated four times using PBMCs obtained from distinct donors to ensure observations were independent of PBMC source.

*Flow Cytometry.* To assess cytokine production, the co-cultures were stimulated with Phorbol Myristate Acetate-Ionomycin (Cat. No. 356150010; Fisher Scientific) and GolgiPlug (Cat. No. 555029; BD Biosciences) for 16 hours. Cells were harvested and single-cell suspensions were stained in two separate antibody panels to assess phenotype. Panel 1: anti-CD3 (Cat. No. 557917; BD Biosciences), anti-CD4 (Cat. No. 563028; BD Biosciences), anti-CD8a (Cat. No. 563821; BioLegend), anti-CD25 (Cat. No. 557741; BD Biosciences), anti-FoxP3 (Cat. No. 14-4776-80; eBioscience), and anti-IL10 (Cat. No. 130-096-043; Miltenyi Biotec). Panel 2: anti-CD3 (Cat. No. 557917; BD Biosciences), anti-CD4 (Cat. No. 563028; BD Biosciences), anti-CD8a (Cat. No. 563821; BioLegend), anti-CD69 (Cat. No. 560737; BD Biosciences), anti-INFγ (Cat. No. 560371; BD Biosciences), anti-IL4 (Cat. No. 130-091-647; Miltenyi Biotec), anti-IL17A (Cat. No. 17-7179-42; eBioscience), and anti-IL22 (Cat. No. 25-7229-42; eBioscience). Cells were permeabilized by either Cytofix/Cytoperm™(Cat. No. 554714; BD Bioscience) or Fixation/ Permeabilization (Cat. No. 00-5523-00; Affymatrix eBioscience). Upon staining, live T-cells were gated as CD3^+^ CD4^+^ or CD3^+^ CD8^+^ cells. Activated T-cells were surface stained CD69hi. Among the CD4^+^ T-cell population, subpopulations were defined as Th1: IFNγ^+^, Th2: IL-4^+^, Th17: IL-17A^+^, Th22: IL17A^-^ and IL-22^+^, and Treg: CD25hi and FoxP3hi. CD8^+^ T-cells subpopulations were defined as Tc1: IFNγ^+^, Tc2: IL-4^+^, and Tc17: IL-17A^+^. Stained cells were assayed via flow cytometry on a BD LSR II (BD Biosciences).

*Cytometric Bead Array*. Prior to addition of PMA/Gplug, 100 uL of cell-free supernatant was removed from each co-culture and centrifuged for 1 minute at 3000 rpm. Cytokine secretion was measured using a cytometric bead array (BD Biosciences) and the concentration of IL-4, IL-5, IL-13, and were determined according to the manufacturer’s guidelines. Data was acquired by flow cytometry on a BD LSR II (BD Biosciences) and data analysis was performed using the proprietary FCAP Array analysis software (BD Biosciences).

**Statistical analysis**

*Microbial, Metagenomic, and Metabolomic Analysis.* Analysis was performed using QIIME v1.8.0 and the R statistical environment ([4](#_ENREF_4), [15](#_ENREF_15)). Shannon’s Diversity and Faith’s Phylogenetic Diversity were calculated using QIIME v 1.8.0 and two-tailed t-tests were performed to identify significant between group differences (e.g. UC vs. Healthy) ([4](#_ENREF_4)). Weighted UniFrac, Canberra, and Bray-Curtis distance matrices were generated using QIIME v 1.8.0 and visualized via NMDS in the R statistical environment using the *vegan* package ([16](#_ENREF_16), [17](#_ENREF_17)). For PhyloChip data, fluorescent intensities were log-normalized prior to calculating Canberra distances. Permutational multivariate analysis of variance (PERMANOVA) using calculated distance matrices was used to determine relationships between existing metadata (i.e. Health Status or Ethnicity) and bacterial, fungal, metagenome, or metabolome composition using the a*donis* function found in *vegan* ([17](#_ENREF_17)). Hierarchical cluster analysis combined with multi-scale, bootstrap resampling was performed using the *pvclust* package in R with 1000 bootstrap replications ([18](#_ENREF_18)). Correlation between distances matrices was calculated using the *mantel* function found in *vegan* ([17](#_ENREF_17)). To identify significantly enriched or depleted bacterial OTUs, fungal OTUs, and KEGG pathways between relevant sample groups (e.g. UC vs. Healthy), the three-model approach described by Romero *et al.* was applied ([19](#_ENREF_19)). Briefly, three linear mixed-effect regression models (negative binomial, zero-inflated negative bionomial, and Poisson) were independently fit to each observation (i.e. OTU or KEGG pathway) and the model with lowest Akaike Information Criterion (AIC) was retained. P-values were computed for only the best-fit models (i.e. those that minimized AIC). To account for false discovery, q-values were calculated based on the computed p-values. For PhyloChip data, significantly enriched or depleted OTUs were determined by applying a two-tailed t-test to log-normalized fluorescent intensities. To identify significantly enriched or depleted fecal metabolites, log-normalized relative concentrations were compared using a Welch’s t-test.

*Comparison of Clinical Measures of Disease Severity.* Clinical measures of disease severity (i.e. SCCA, number of extra-colonic manifestations, number of diagnosed first- and second-degree relatives, and years since diagnosis were compared between UC-MCS by a Kruskal-Wallis Test followed by pairwise tw-tailed Dun’s Test.

*Analysis of T-cell Subsets.* Because the T-cell assay described above was repeated four separate times using PBMCs from four different PBMC donors, a linear mixed effects model was applied using the *lme4* package in R to identify significant differences in the abundance of induced T-cell subpopulations based on sample group (i.e. UC-MCS) while accounting for potential variation introduced due to PBMC source (i.e. donor) ([20](#_ENREF_20)). The following linear mixed effects models were applied to identify changes due to health status (Healthy vs. UC) and UC-MCS (Healthy vs. MCS1, MCS2, MCS3, MCS4) respectively:

Y ~ β(EXP_GROUP) + μ(DONOR) + μ(SAMPLE) + ε

Y ~ β(MCS) + μ(DONOR) + μ(SAMPLE) + ε

Where Y = a measured, dependent variable such as Th1 abundance, EXP_GROUP = health status (Healthy or UC), MCS = microbial community state (Healthy, MCS1, MCS2, MCS3, or MCS4), DONOR = PBMC donor source (Donor #1 to #4), and SAMPLE = fecal sample study participant.

**SUPPLEMENTARY REFERENCES**

1. **Walmsley RS, Ayres RC, Pounder RE, Allan RN.** 1998. A simple clinical colitis activity index. Gut **43:**29-32.

2. **Caporaso JG, Lauber CL, Walters WA, Berg-Lyons D, Huntley J, Fierer N, Owens SM, Betley J, Fraser L, Bauer M, Gormley N, Gilbert JA, Smith G, Knight R.** 2012. Ultra-high-throughput microbial community analysis on the Illumina HiSeq and MiSeq platforms. ISME J **6:**1621-1624.

3. **Magoc T, Salzberg SL.** 2011. FLASH: fast length adjustment of short reads to improve genome assemblies. Bioinformatics **27:**2957-2963.

4. **Caporaso JG, Kuczynski J, Stombaugh J, Bittinger K, Bushman FD, Costello EK, Fierer N, Pena AG, Goodrich JK, Gordon JI, Huttley GA, Kelley ST, Knights D, Koenig JE, Ley RE, Lozupone CA, McDonald D, Muegge BD, Pirrung M, Reeder J, Sevinsky JR, Turnbaugh PJ, Walters WA, Widmann J, Yatsunenko T, Zaneveld J, Knight R.** 2010. QIIME allows analysis of high-throughput community sequencing data. Nat Methods **7:**335-336.

5. **Edgar RC.** 2010. Search and clustering orders of magnitude faster than BLAST. Bioinformatics **26:**2460-2461.

6. **DeSantis TZ, Hugenholtz P, Larsen N, Rojas M, Brodie EL, Keller K, Huber T, Dalevi D, Hu P, Andersen GL.** 2006. Greengenes, a chimera-checked 16S rRNA gene database and workbench compatible with ARB. Appl Environ Microbiol **72:**5069-5072.

7. **Caporaso JG, Bittinger K, Bushman FD, DeSantis TZ, Andersen GL, Knight R.** 2010. PyNAST: a flexible tool for aligning sequences to a template alignment. Bioinformatics **26:**266-267.

8. **Haas BJ, Gevers D, Earl AM, Feldgarden M, Ward DV, Giannoukos G, Ciulla D, Tabbaa D, Highlander SK, Sodergren E, Methe B, DeSantis TZ, Human Microbiome C, Petrosino JF, Knight R, Birren BW.** 2011. Chimeric 16S rRNA sequence formation and detection in Sanger and 454-pyrosequenced PCR amplicons. Genome Res **21:**494-504.

9. **Price MN, Dehal PS, Arkin AP.** 2009. FastTree: computing large minimum evolution trees with profiles instead of a distance matrix. Mol Biol Evol **26:**1641-1650.

10. **Koljalg U, Nilsson RH, Abarenkov K, Tedersoo L, Taylor AF, Bahram M, Bates ST, Bruns TD, Bengtsson-Palme J, Callaghan TM, Douglas B, Drenkhan T, Eberhardt U, Duenas M, Grebenc T, Griffith GW, Hartmann M, Kirk PM, Kohout P, Larsson E, Lindahl BD, Lucking R, Martin MP, Matheny PB, Nguyen NH, Niskanen T, Oja J, Peay KG, Peintner U, Peterson M, Poldmaa K, Saag L, Saar I, Schussler A, Scott JA, Senes C, Smith ME, Suija A, Taylor DL, Telleria MT, Weiss M, Larsson KH.** 2013. Towards a unified paradigm for sequence-based identification of fungi. Mol Ecol **22:**5271-5277.

11. **Bengtsson-Palme J, Ryberg M, Hartmann M, Branco S, Wang Z, Godhe A, De Wit P, Sánchez-García M, Ebersberger I, de Sousa F, Amend A, Jumpponen A, Unterseher M, Kristiansson E, Abarenkov K, Bertrand YJK, Sanli K, Eriksson KM, Vik U, Veldre V, Nilsson RH.** 2013. Improved software detection and extraction of ITS1 and ITS2 from ribosomal ITS sequences of fungi and other eukaryotes for analysis of environmental sequencing data. Methods in Ecology and Evolution **4:**914-919.

12. **Cox MJ, Allgaier M, Taylor B, Baek MS, Huang YJ, Daly RA, Karaoz U, Andersen GL, Brown R, Fujimura KE, Wu B, Tran D, Koff J, Kleinhenz ME, Nielson D, Brodie EL, Lynch SV.** 2010. Airway microbiota and pathogen abundance in age-stratified cystic fibrosis patients. PLoS One **5:**e11044.

13. **Hazen TC, Dubinsky EA, DeSantis TZ, Andersen GL, Piceno YM, Singh N, Jansson JK, Probst A, Borglin SE, Fortney JL, Stringfellow WT, Bill M, Conrad ME, Tom LM, Chavarria KL, Alusi TR, Lamendella R, Joyner DC, Spier C, Baelum J, Auer M, Zemla ML, Chakraborty R, Sonnenthal EL, D'Haeseleer P, Holman HY, Osman S, Lu Z, Van Nostrand JD, Deng Y, Zhou J, Mason OU.** 2010. Deep-sea oil plume enriches indigenous oil-degrading bacteria. Science **330:**204-208.

14. **Langille MG, Zaneveld J, Caporaso JG, McDonald D, Knights D, Reyes JA, Clemente JC, Burkepile DE, Vega Thurber RL, Knight R, Beiko RG, Huttenhower C.** 2013. Predictive functional profiling of microbial communities using 16S rRNA marker gene sequences. Nat Biotechnol **31:**814-821.

15. **Team RC.** 2015. R: A Language and Environment for Statistical Computing.

16. **Lozupone C, Knight R.** 2005. UniFrac: a new phylogenetic method for comparing microbial communities. Appl Environ Microbiol **71:**8228-8235.

17. **Jari Oksanen FGB, Roeland Kindt, Pierre Legendre, Peter R. Minchin, R. B. O'Hara, Gavin L. Simpson, Peter Solymos, M. Henry H. Stevens, and Helene Wagner.** 2015. vegan: Community Ecology Package.

18. **Suzuki R, Shimodaira H.** 2006. Pvclust: an R package for assessing the uncertainty in hierarchical clustering. Bioinformatics **22:**1540-1542.

19. **Romero R, Hassan SS, Gajer P, Tarca AL, Fadrosh DW, Nikita L, Galuppi M, Lamont RF, Chaemsaithong P, Miranda J, Chaiworapongsa T, Ravel J.** 2014. The composition and stability of the vaginal microbiota of normal pregnant women is different from that of non-pregnant women. Microbiome **2:**4.

20. **Bates D. M. MM, Bolker B. M., Walker S. C.** 2015. Fitting Linear Mixed-Effects Models Using {me4. Journal of Statistical Software **67**.
